# Causal association between gastrointestinal diseases and coronary artery disease: a bidirectional Mendelian randomization study

**DOI:** 10.3389/fendo.2024.1458196

**Published:** 2024-10-15

**Authors:** Zhuoxi Wang, Jifang Ban, Yabin Zhou, Rui Qie

**Affiliations:** ^1^ First Clinical Medical School, Heilongjiang University of Chinese Medicine, Harbin, Heilongjiang, China; ^2^ Department of Cardiovascular, First Affiliated Hospital of Heilongjiang University of Chinese Medicine, Harbin, Heilongjiang, China; ^3^ Preventive Treatment Center, First Affiliated Hospital of Heilongjiang University of Chinese Medicine, Harbin, Heilongjiang, China

**Keywords:** causal inference, coronary artery disease, gastrointestinal diseases, Mendelian randomization, statistical method

## Abstract

**Background:**

Coronary artery disease (CAD) has been a dominating reason of mortality globally due to its complexity of etiology. A variety of gastrointestinal disorders (GDs) have been accounted to be related to CAD. Thus, this study aims to determine their causal relationship by two-sample Mendelian randomization (MR) analysis.

**Methods:**

Single-nucleotide polymorphisms (SNPs) relevant to 22 GDs were employed as instrumental variables from the genome-wide association summary (GWAS) datasets. Genetic associations with CAD and HF were acquired from UK Biobank, FinnGen, and other GWAS studies. We conducted a univariable MR (UVMR) analysis followed by a meta-analysis. A multivariable MR (MVMR) analysis was then performed with smoking and body mass index (BMI) as justifications. Also, a bi-directional MR analysis was leveraged to verify the reverse causal correlations.

**Results:**

Generally, UVMR analyses separately observed the causal effects of GDs on CAD and HF. Genetic liability to gastroesophageal reflux disease displayed a positive association with both CAD (OR=1.19; 95%CI: 1.01-1.41) and HF (OR=1.22; 95%CI: 1.00-1.49) risk; genetic liability to celiac disease separately attributed to CAD (OR=1.02; 95%CI: 1.01-1.03) and HF (OR=1.01; 95%CI: 1.00-1.02), which also maintained after MVMR analysis. Besides, we observed mutually causal associations between CAD and celiac disease.

**Conclusion:**

Our work suggested that genetic susceptibility to some GDs might causally increase the risk of CAD and HF, emphasizing the importance of preventing CAD in patients with GDs.

## 1 Introduction

By the 2020s, coronary artery disease (CAD) was found to affect approximately 197 million patients and 9.1 million deaths, accounting for 16.1% of all deaths globally, which is the most common cardiovascular disease caused by the formation of atherosclerosis due to the accumulation of fat or inflammatory factors in the coronary arteries (1, 2). The plaque may contribute to cardiac ischemia or infarction, usually called ischemic heart disease (IHD), and develop into acute coronary syndrome (ACS), heart failure (HF), and sudden death. The risk factors for CAD progression and exacerbation are interactive rather than independent, including poor lifestyle habits such as smoking, alcohol consumption, sedentary behaviour, and mental stress, as well as multiple risk factors such as obesity, type 2 diabetes, hypertension, and metabolic syndrome (3–5). In addition, genetic and family history cannot be ignored. Considering the intricate pathogenesis of CAD, screening causal risk factors and preventing them is imperative, which is meaningful for attenuating the burden of related public health services (6).

Accumulating evidence indicates that the dysregulation of gut microbiota could play a role in cardiac diseases, with a focus on CAD and HF, and there are significant alterations between the structure and composition of the gut microbiota in CAD patients compared with controls. Classic microbiota-associated metabolites have been found to affect the progression of cardiac disease, such as trimethylamine-N-oxides (TMAO), short-chain fatty acids (SCFAs), lipopolysaccharide (LPS) and secondary bile acids (7–9). However, the sample size of the studies is too small, and there is a lack of control for essential covariates such as drugs, diet, and complications. On the other hand, the gut microbiome is so large and variable that the relationship between the changes in gut microbiota and its metabolites and cardiovascular disease cannot be determined (10). Therefore, this study returned to specific digestive disorders to see if they have a direct causal relationship with CAD and explore the gut-heart axis clinically.

Currently, gastrointestinal diseases (GDs) are high-profile, and accumulating evidence suggests that they are responsible for multi-system disorders, such as Alzheimer’s disease in the enteric nervous system (11), depression of psychological aspect (12), metabolic syndrome (13), and cardiovascular diseases (14). Population-based studies from epidemiology have observed that coronary atherosclerosis might be caused by nonalcoholic fatty liver disease and liver cirrhosis (15, 16); gastroesophageal reflux disease (GERD) has been reported to cause angina and cardiac rhythm disturbances (17); ulcerative colitis to result in increased morbidity for CAD events (18). However, these observational causal connections are only sometimes consistent, particularly in the case of cardio-hepatic comorbidity. For example, a population-based study found that probably because the gluten-free recipe lowered blood pressure and cholesterol levels, celiac disease decreased the onset of myocardial infarction slightly (19).

The emerging MR analysis, supported by large-scale genome-wide association studies (GWAS) statistic data, has an advantage in identifying the etiogenic inference between risk agents and disease events by using genetic loci as instrumental variables (IVs) for exposure (20). Unlike conventional epidemiological methods, the MR approach exhibits merit in averting bias with confusion as alleles are assorted randomly when fertilizing instead of being confused by postnatal and self-developed factors (21). Also, MR analysis holds back an inverse relationship due to the lack of modification of germline genotype by disease (22). Although published MR studies have reported the cause of several GDs, including GERD (23), celiac disease (24), and inflammatory bowel disease (25), on the risk of CAD, these shreds of evidence were largely independent.

In this article, we aimed to comprehensively inspect the associations of GDs with CAD by performing a two-sample MR study in both directions, followed by a meta-analysis. Additionally, according to updated data from the Framingham Heart Study, CAD is a major risk factor for HF (26). Thus, we examined the relationships between GDs and HF as supplementary evidence. Finally, we carried out a multivariable MR (MVMR) analysis to justify conventional predisposing agents.

## 2 Study design and methods

### 2.1 Study design

**Figure 1:**
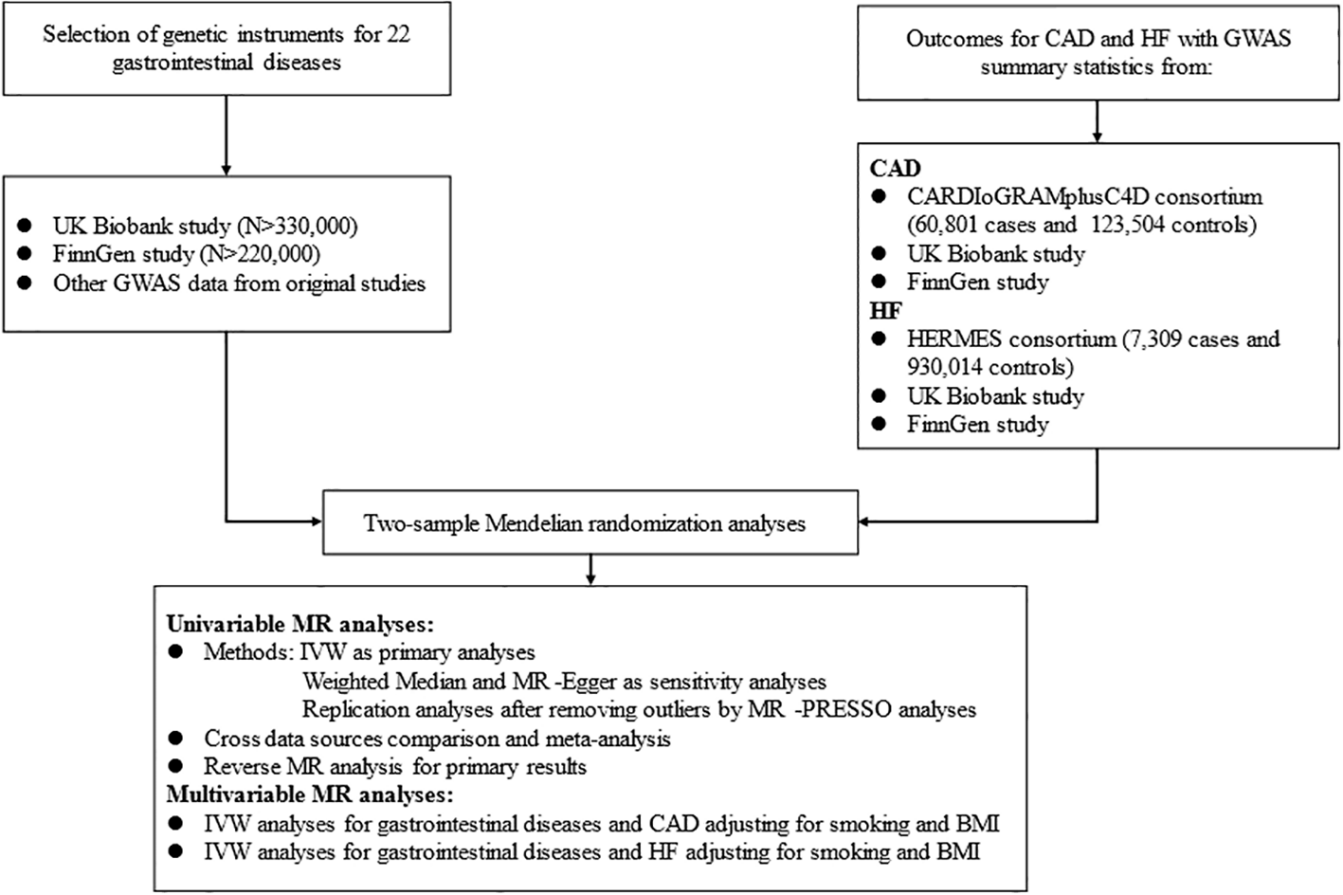
Flow diagram of the data acquisition and analytic process for the study.

This MR analysis was designed to examine the causal effect of common GDs on the risk of CAD and HF (**Figure 1**). We first performed a univariable MR (UVMR) analysis using 22 kinds of GDs and CAD as well as HF GWAS summary data of European populations, publicly available in the UK Biobank (UKB) study (27), FinnGen study, and other prominent international consortia. Then, we combined the findings from the above MR studies with a meta-analysis. We also conducted a bi-directional MR analysis to estimate the inverse causation. Finally, we verified the causal factors irrelevant to traditional risk elements by MVMR analysis, including smoking and body mass index (BMI).

### 2.2 GWAS data for gastrointestinal diseases

We collected 22 GDs and assigned them to four broad location groups, including six upper GDs, six lower GDs, nine hepato-biliary and pancreas diseases, and others. Detailed data sources information for GDs was collected in **Additional File 1: Supplementary Table 1**.

The criterion for selecting eligible IVs for GDs needs to be met the following three hypotheses: (1) related to the exposure, namely relevance hypothesis; (2) uncorrelated with any confounding factors, namely independence hypothesis; (3) unconcerned with the outcome under exposure conditions, namely exclusion restriction hypothesis (20). Therefore, single-nucleotide polymorphisms (SNPs) were selected through a series of steps as valid IVs. First, due to the limitation of numbers of SNPs satisfying the significant genome-wide, we appropriately widened the associated boundary with *p* < 1 × 10^–6^ to obtain top associated SNPs, except for GERD using *P* < 5×10^-8^. Second, those SNPs with linkage disequilibrium (LD) (r ^2^ > 0.001 and clump distance < 10 MB) were eliminated to acquire top independent SNPs. Meanwhile, we calculated the *F* statistics of individual SNP to prevent weak IVs, which was greater than the threshold of 10 (20). Then, we extracted the acquired SNPs associated with exposure from the outcome datasets. For SNPs unavailable in CAD outcomes, we searched proxies in high LD (r^2^ > 0.8) in the European reference panel of the 1000 Genomes Project (28), and we discarded those still unavailable through proxy. Finally, we coordinated the SNP alleles, which were associated not only with exposure but also with outcome. The procedures for selecting IVs in reverse MR analysis were the same. Applicable SNPs used in this study are shown in **Additional File 1: Supplementary Tables 2–4**.

### 2.3 GWAS data for CAD

The GWAS statistical data for CAD was obtained from the Coronary Artery Disease Genome-wide Replication and Meta-analysis plus The Coronary Artery Disease Genetics (CARDIoGRAMplusC4D) consortium, comprising 6 0801 cases which were identified as an inclusive diagnosis of myocardial infarction, acute coronary syndrome, chronic stable angina, or coronary stenosis >50% and 12 3504 controls from 48 individual studies (29). The summarized statistical data for HF in European ancestry was acquired from the Heart Failure Molecular Epidemiology for Therapeutic Targets (HERMES) consortium, including 47 309 samples and 930 014 controls (30). We further collected genetic information for CAD and HF from the UKB and FinnGen studies to conduct replication analyses, respectively.

The UKB research is a large-scale, promising cohort study with more than 500,000 subjects recruited from individual centers across the UK from 2006 to 2010. Summary level data for genetically related CAD in this study were available from UKB, involving 17655 cases defined by the codes I21 and I22 from the International Classification of Diseases version 10 (ICD-10). We further collected the European GWAS statistics for HF in UKB with the code I50 in ICD-10, comprising 1088 cases and 360 106 controls launched by the Neale Lab (27).

The FinnGen consortium aims to study genetic information with 30,952 samples and 187,840 controls from Finnish ancestry. We used the fifth release of the GWAS data on CAD and HF, diagnosed by the ICD-10 (31). Detailed information on data sources and SNPs used as IVs are displayed in **Additional File 1: Supplementary Tables 1–4**.

### 2.4 Statistical analysis

In this MR study, we applied UVMR analysis to evaluate the causal role of genetic liability to GDs on the risk of CAD and HF. We employed the inverse variance weighted (IVW) as the primary analysis, which produces the most accurate estimates but is liable to be biased by pleiotropy due to the assumption that all genetic instruments are valid (32). We also used weighted median and MR-Egger methods to conduct sensitivity analyses, purposing to inspect the stability of the causality (33, 34). The weighted median assumes that at least 50% of the SNPs are valid and provide the weight of estimates, while MR-Egger regression assumes that all SNPs are invalid. MR Pleiotropy Residual Sum and Outlier (MR-PRESSO) method can detect underlying horizontal pleiotropy (35) and correct for potential outliers contributing to heterogeneity across SNPs’ estimates (36). We replicated primary and sensitivity analyses after discarding the outliers if significant pleiotropy was detected. The MR results were presented as odds ratio (OR) and 95% confidence interval (CI). Finally, we conducted a meta-analysis of IVW estimates to identify the combined causality of GDs on CAD and HF from diverse sources. When there was pleiotropy, we selected the later MR results for the meta-analysis. Based on the *I* ^2^ statistic, we used a fixed effect for *I* ^2^ < 50%; otherwise, a random effect for *I* ^2^ > 50% (37). The effects of meta-analysis were considered as the final causal references. Meanwhile, we were also concerned about other MR results that were only significant in a particular database. Furthermore, we conducted a reverse MR analysis if the causality was detected in the forward analysis, and the final results were also evaluated with a meta-analysis.

We also performed the Cochran Q test and generated a funnel plot to appraise the heterogeneity and a leave-one-out (LOO) analysis to determine whether a specific SNP drove the pooled MR estimates (35). Besides, we adopted the Steiger directional test to determine the positive bi-directional causalities in forward MR analysis (38). Moreover, we applied the Benjamini-Hochberg approach to adjust for multiple testing. All analyses were completed using “TwoSampleMR” (39) and “MRPRESSO” R packages in RStudio Version 4.2.3 (35).

## 3 Results

### 3.1 Gastrointestinal diseases and CAD

**Figure 2:**
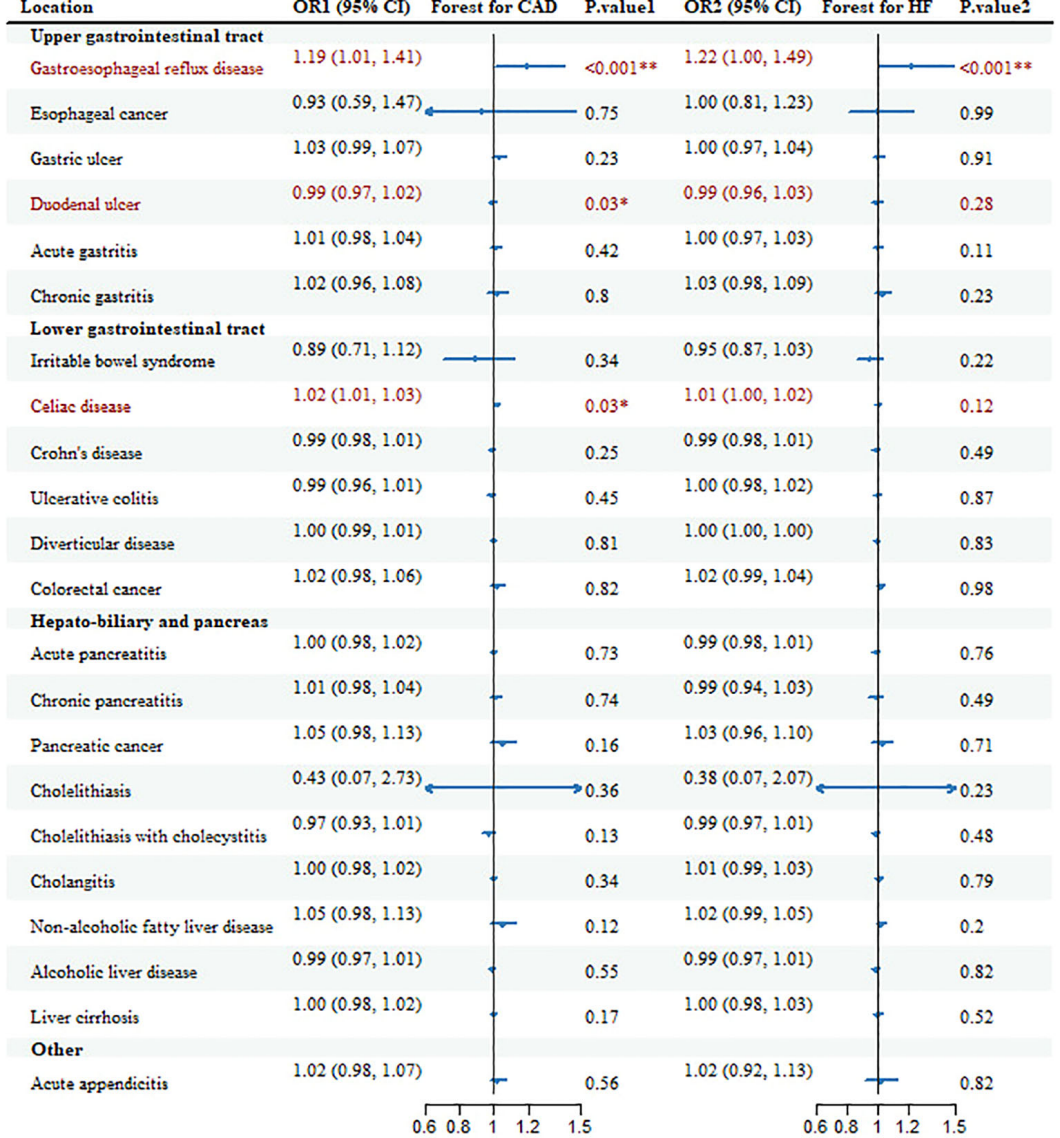
Forest plots and combined causality for the association of gastrointestinal diseases with CAD and HF. *p < 0.05 for combined effects; **p < 0.05 after adjusting for multiple testing.

Hereditary susceptibility to 3 GDs were increasingly associated with risk of CAD in meta-analysis (**Table 1**, **Figure 2**). For 1-unit increment in log-transformed OR of GERD and celiac disease, the combined OR was separately 1.19 (95%CI: 1.01-1.41; *p*_meta_=0.0001) and 1.02 (95% CI: 1.01-1.03; *p*_meta_=0.0284) for CAD risk, together duodenal ulcer with the same directional association (OR=0.99; 95%CI: 0.97-1.02; *p*_meta_=0.0307).

**Table 1:** Combined causal relationship between 22 gastrointestinal diseases and CAD or HF.

Exposures for GDs	Combined results for IVW							
	CAD				HF			
	Data sources	OR (95%CI)	*P* value	*I* ^2^ (%)	Data sources	OR (95%CI)	*P* value	*I* ^2^ (%)
Upper gastrointestinal tract								
Gastroesophageal reflux disease	CARDIoGRAMplusC4D+UKB+FinnGen	**1.19 (1.01, 1.41)**	**0.0001**	97	HERMES+UKB+FinnGen	**1.22 (1.00, 1.49)**	**0.0001**	98
Esophageal cancer	CARDIoGRAMplusC4D+UKB+FinnGen	0.93 (0.59, 1.47)	0.75	0	HERMES+UKB+FinnGen	1.00 (0.81, 1.23)	0.99	0
Gastric ulcer	CARDIoGRAMplusC4D+UKB+FinnGen	1.03 (0.99, 1.07)	0.23	73	HERMES+UKB+FinnGen	1.00 (0.97, 1.04)	0.91	0
Duodenal ulcer	CARDIoGRAMplusC4D+UKB+FinnGen	**0.99 (0.97, 1.02)**	**0.03**	0	HERMES+UKB+FinnGen	0.99 (0.96, 1.03)	0.28	0
Acute gastritis	CARDIoGRAMplusC4D+UKB+FinnGen	1.01 (0.98, 1.04)	0.42	0	HERMES+UKB+FinnGen	1.00 (0.97, 1.03)	0.11	0
Chronic gastritis	CARDIoGRAMplusC4D+UKB+FinnGen	1.02 (0.96, 1.08)	0.80	48	HERMES+UKB+FinnGen	1.03 (0.98, 1.09)	0.23	65
Lower gastrointestinal tract								
Irritable bowel syndrome	CARDIoGRAMplusC4D+UKB+FinnGen	0.89 (0.71, 1.12)	0.34	0	HERMES+UKB+FinnGen	0.95 (0.87, 1.03)	0.22	0
Celiac disease	CARDIoGRAMplusC4D+UKB+FinnGen	**1.02 (1.01, 1.03)**	**0.03**	48	HERMES+UKB+FinnGen	**1.01 (1.00, 1.02)**	**0.12**	0
Crohn’s disease	CARDIoGRAMplusC4D+UKB+FinnGen	0.99 (0.98, 1.01)	0.25	71	HERMES+UKB+FinnGen	0.99 (0.98, 1.01)	0.49	66
Ulcerative colitis	CARDIoGRAMplusC4D+UKB+FinnGen	0.99 (0.96, 1.01)	0.45	28	HERMES+UKB+FinnGen	1.00 (0.98, 1.02)	0.87	27
Diverticular disease	CARDIoGRAMplusC4D+UKB+FinnGen	1.00 (0.99, 1.01)	0.81	66	HERMES+UKB+FinnGen	1.00 (1.00, 1.00)	0.83	0
Colorectal cancer	CARDIoGRAMplusC4D+UKB+FinnGen	1.02 (0.98, 1.06)	0.82	0	HERMES+UKB+FinnGen	1.02 (0.99, 1.04)	0.98	0
Hepato-biliary and pancreas								
Acute pancreatitis	CARDIoGRAMplusC4D+UKB+FinnGen	1.00 (0.98, 1.02)	0.73	0	HERMES+UKB+FinnGen	0.99 (0.98, 1.01)	0.76	15
Chronic pancreatitis	CARDIoGRAMplusC4D+UKB+FinnGen	1.01 (0.98, 1.04)	0.74	0	HERMES+UKB+FinnGen	0.99 (0.94, 1.03)	0.49	80
Pancreatic cancer	CARDIoGRAMplusC4D+UKB+FinnGen	1.05 (0.98, 1.13)	0.16	82	HERMES+UKB+FinnGen	1.03 (0.96, 1.10)	0.71	0
Cholelithiasis	CARDIoGRAMplusC4D+UKB+FinnGen	0.43 (0.07, 2.73)	0.36	77	HERMES+UKB+FinnGen	0.38 (0.07, 2.07)	0.23	80
Cholelithiasis with cholecystitis	CARDIoGRAMplusC4D+UKB+FinnGen	0.97 (0.93, 1.01)	0.13	90	HERMES+UKB+FinnGen	0.99 (0.97, 1.01)	0.48	57
Cholangitis	CARDIoGRAMplusC4D+UKB+FinnGen	1.00 (0.98, 1.02)	0.34	0	HERMES+UKB+FinnGen	1.01 (0.99, 1.03)	0.79	0
Non-alcoholic fatty liver disease	CARDIoGRAMplusC4D+UKB+FinnGen	1.05 (0.98, 1.13)	0.12	92	HERMES+UKB+FinnGen	1.02 (0.99, 1.05)	0.20	74
Alcoholic liver disease	CARDIoGRAMplusC4D+UKB+FinnGen	0.99 (0.97, 1.01)	0.55	0	HERMES+UKB+FinnGen	0.99 (0.97, 1.01)	0.82	27
Liver cirrhosis	CARDIoGRAMplusC4D+UKB+FinnGen	1.00 (0.98, 1.02)	0.17	0	HERMES+UKB+FinnGen	1.00 (0.98, 1.03)	0.52	0
Other								
Acute appendicitis	CARDIoGRAMplusC4D+UKB+FinnGen	1.02 (0.98, 1.07)	0.56	0	HERMES+UKB+FinnGen	1.02 (0.92, 1.13)	0.82	0

Bold values mean p < 0.05.

Additionally, there were other causal references of GDs with CAD in some databases, but the causality lost significance after the combination. Genetic liability to gastric ulcer with OR=1.06 (95%CI: 1.00-1.12; *p*=0.047), Crohn’s disease with OR=0.979 (95%CI: 0.963-0.997; *p*=0.02) and NAFLD with OR=1.14 (95%CI: 1.08-1.20; *p*=1.08E-06) was positively associated with CAD only in CARDIoGRAMplusC4D; pancreatic cancer with OR=1.10 (95%CI: 1.03-1.17; *p*=4.65E-03) and cholelithiasis with OR=0.05 (95%CI: 0.01-0.37; *p*=3.20E-03) only in FinnGen. All these associations, only the risk of GERD on CAD, remained significant after multiple tests (**Additional File 1: Supplementary Table 5**).

The causal effects of GDs on CAD were in accordance with primary results in sensitivity analyses in the same direction (**Additional File 1: Supplementary Table 6**). We detected heterogeneity in several gastrointestinal outcomes. We also observed horizontal pleiotropy in the MR-Egger test for cholelithiasis and NAFLD. As for MR-PRESSO analysis, we identified one to 16 outliers in all three data sources, mainly for celiac disease, cholelithiasis, and NAFLD. These associations survived but with smaller estimates and more rigorous CIs after removing outliers except for that for cholelithiasis with no substantial risk of CAD.

Reversely, no causal associations of CAD on GDs were detected, including GERD (OR=1.02; 95%CI: 0.99-1.04; *p*_meta=_0.1356) and duodenal ulcer (OR, 1.00; 95%CI, 1.00-1.00; *p*_meta=_0.2125) (**Additional File 1: Supplementary Table 7**). Interestingly, CAD appeared to affect the onset of celiac disease (OR=1.24; 95%CI: 1.02-1.50; *p*_meta=_0.0279), implying a bidirectional causal reference, which was also identified in Steiger’s directional test (**Additional File 1: Supplementary Table 8**). The above results remained stable in sensitivity analyses and survived after adjustment in MVMR analysis (**Additional File 1: Supplementary Table 9**).

### 3.2 Gastrointestinal diseases and HF

Genetic susceptibility to 2 GDs was correlated to the risk of HF (**Table 1**, **Figure 2**). With genetic prediction, per-unit increment in log-transformed OR of GERD was highly associated with HF (OR=1.22; 95% CI: 1.00-1.49; *p*_meta_=0.0001) and celiac disease (OR=1.01; 95% CI: 1.00-1.02; *p*_meta_=0.12), but the significant effect for celiac disease did not maintain in multiple testing. Meanwhile, there were associations for Crohn’s disease (OR=0.97; 95% CI: 0.94-0.99; *p*=0.02), chronic pancreatitis (OR=0.94; 95%CI: 0.90-0.98; *p*=2.50E-03), cholelithiasis (OR=0.05; 95%CI: 0.01-0.33; *p*=1.93E-03), cholelithiasis with cholecystitis (OR=0.96; 95%CI: 0.92-1.00; *p*=0.03) with HF in FinnGen, and NAFLD (OR=1.05; 95%CI: 1.00-1.09; *p*=0.02) in HERMES.

These causal associations remained directionally unitive in sensitivity analyses; however, we still detected heterogeneity and horizontal pleiotropy in MR-Egger for some outcomes, such as GERD in HERMES. Besides, we detected a few outliers in MR-PRESSO in most consequences; despite smaller effect sizes, the causal effects survived after removing these outliers (**Additional File 1: Supplementary Table 10**).

We further detected bi-directional MR analysis of HF on these GDs, and no evidence suggesting a causal effect for GERD (OR=1.07; 95%CI: 0.97-1.19; *p*_meta=_0.1959) and celiac disease (OR=1.21; 95%CI: 0.84-1.75; *p*_meta=_0.2662). Besides, we did not observe horizontal pleiotropy except for NAFLD (**Additional File 1: Supplementary Table 11**).

In short, we ensured the reliability of UVMR analyses in the following areas. First, the selected IVs were valid as the *F* statistics were above 10, except for esophageal cancer, irritable bowel syndrome, and cholelithiasis, suggesting bias from weak instrumentals (**Additional File 1: Supplementary Tables 3, 4**). Second, although there was heterogeneity in Cochran’s Q test and pleiotropy in the MR-Egger examination for some causalities (**Additional File 1: Supplementary Tables 6, 10**), we did not inspect severe asymmetry in funnel plots and significant Egger intercepts in scatter plots after removing the outliers. Further, no single SNP disturbed the combined effect of GDs on CAD and HF in LOO analysis (**Additional File 2: Supplementary Figures 1–18**).

### 3.3 Multivariable MR analysis

We further adjusted for smoking and BMI using MVMR analysis to rule out underlying pleiotropies (**Additional File 1: Supplementary Tables 12, 13**). It turned out that genetic liability to GERD still displayed the strongest association with CAD (OR=1.15; 95%CI: 1.00-1.33; *p*_meta_=0.0001) for smoking and (OR=1.14; 95%CI: 1.00-1.31; *p*_meta_ =0.0001) for BMI, together with HF (OR=1.18; 95%CI: 1.00-1.41; *p*_meta_ =0.0019) for smoking and (OR=1.16; 95%CI: 1.00-1.37; *p*_meta_ =0.0008) for BMI. The causal effect of celiac disease on HF barely reached significance after correcting with smoking (OR=1.01; 95%CI: 1.00-1.02; *p*_meta_=0.2405) and BMI (OR=1.00; 95%CI: 1.00-1.01; *p*_meta_ =0.4030), but acute appendicitis lost the significance, implying that we should treat these causalities with caution.

Intricately, when added to MVMR analysis, BMI exhibited an attractive influence on the association of colorectal cancer and CAD, which made the causality statistically positive (OR=0.9968; 95%CI: 0.9940-0.9996; *p*_meta_=0.0263; beta=-0.0032). Furthermore, smoking also contributed to a significantly increased causal effect of irritable bowel syndrome on HF (OR=0.73; 95%CI: 0.56-0.97; *p*_meta_ =0.0285; beta=-0.3085).

## 4 Discussion

Exploration and illustration of the causal risk factors of GDs on cardiovascular diseases, usually coexisting clinically, can inform the formulation of optimal prevention strategies early. This study estimated the causal association of 22 GDs with CAD and HF by the two-sample MR analysis and verified the results. By analyzing diverse large-scale GWAS datasets using an integrated MR analysis process, we confirmed several genetic liabilities to GDs that exerted a causal role on cardiac outcomes. Meanwhile, we ruled out some of those traditionally regarded as risk factors for CAD.

The results indicated robust relationships between genetic predisposition to GERD and increased risk of CAD and HF independent of adjustment factors for multivariate variables. In addition, there is a bidirectional causal effect of coeliac disease and CAD. A study of genetic populations in the UK over the last ten years found that the incidence of coeliac disease was one of the autoimmune diseases with the most significant increase and was significantly associated with environmental factors, showing a transparent socioeconomic gradient related to Diet, smoking, obesity, air pollution or other currently unrecognised ecological exposures. Surprisingly, this is a reverse socioeconomic gradient, which may be related to increased awareness and detection of the disease in more affluent populations. It is well known that cardiovascular disease is strongly associated with a poor lifestyle. Although some studies have found a decline in smoking prevalence, no corresponding decrease in morbidity has been found, which may be due to a parallel increase in other risk factors, such as obesity, over the same period. Therefore, caution should be exercised in interpreting the bidirectional causal relationship between coeliac disease and CAD, possibly due to shared confounding effects of environmental factors.Also, genetically predicted duodenal ulcer was mild and related to increased risk of CAD, but not HF. Fortunately, we did not find that common malignant gastrointestinal cancers, including gastric, colorectal and pancreatic cancers, may induce heart disease, even though cardiotoxicity, possibly due to the use of anticancer drugs, is identifiable and manageable (40). In addition to these common GDs, enterovirus infections remain a primary contributor to acute gastroenteritis worldwide (41). According to the epidemiological characteristics of the virus, viral infection requires the host to have the opportunity to contact with pathogenic microorganisms, including environmental or dietary exposure, but this is preventable. Therefore, this study did not investigate various viral infections as exposure factors for CAD (42). As for inflammatory bowel disease (IBD), we often refer to it as characterized by chronic and relapsing intestinal inflammation; it is usually defined as either Crohn’s disease or ulcerative colitis and irritable bowel syndrome that overlaps with symptoms associated with IBD. Although we found that Crohn’s disease increased the risk of CAD and HF in Finngen, respectively, the pooled MR results did not detect such a strong association, suggesting that we may have bias and cannot ignore the effect of IBD on heart disease.

As one of the most common GDs, GERD traditionally manifests as heartburn and acid regurgitation, yet it usually presents with atypical symptoms, such as epigastric pain and chest pain, that mimic those of CAD (43); thus, it is difficult to distinguish between the two and contribute to misdiagnosis. To date, increasing epidemiological proofs observe a concurrence of GERD and CAD due to the utilization of proton pump inhibitors potentially (44). Therefore, early prevention of CAD in patients with GERD is necessary. Consistent with Sun et al. (23), we confirmed a causal correlation between a hereditary disposition to GERD and a risky onset of CAD and did not find any causal presence of CAD on GERD through a bidirectional MR analysis. Additionally, in line with a nationwide cohort study (45), we discovered that genetic liability to GERD increased the risk of HF. Besides, these two causalities survived after multiple testing and MVMR analysis.

We also detected an increased risk between genetic liability to celiac disease and CAD, verified in epidemiology (46). Although the causality was not maintained in multiple testing and MVMR analysis, indicating that the causal role of celiac disease on CAD might be biased by smoking and BMI traits, our results were still more persuasive as using 56, 57, and 52 SNPs from three diverse GWAS data sources separately than those of Huang based on 15 SNPs from only one GWAS dataset (24). Notably, we observed a bidirectional relationship between celiac disease and CAD, which might attributed to the shared pathogenesis, such as anabatic systemic inflammation, triggering celiac disease in individuals with CAD (47). Moreover, the inverse causality was reserved after adjusting for smoking and BMI, and the effect sizes were even more significant than those in forward MR analysis. Overall, it is of importance clinically to promote the identification and therapy of celiac disease in CAD patients.

Furthermore, we observed a slightly increased risk of duodenal ulcer on CAD, but the causality may be false positive biased by heterogeneity from different GWAS data sources. Inversely, the finding from a prospective study was that patients with CAD were highly associated with duodenal ulcers due to the use of aspirin (48). Given this controversy in epidemiology and the slight significance of this study, the relationship between duodenal ulcer and CAD still needs to be explored.

We also found that genetic liability to celiac disease is causally associated with HF with a very slight significance, and the point estimate was mildly above 1, suggesting that the causality might be expected just by chance. Unexpectedly, celiac disease was seemingly relevant with a diminished encumbrance of traditional risk factors for cardiovascular diseases, such as higher BMI and incidence of type 2 diabetes, and a smaller likelihood of smoking (49), which explained why the effect sizes did not differ much before and after adjustment.

It is well known that smoking, alcohol consumption and obesity are independent high-risk factors for cardiovascular diseases (50, 51). Therefore, when considering the direct causal effect of GDs on CAD, the confounding effects of smoking and alcohol consumption should be adjusted. The results of MVMR suggest that the causal effects of GERD on CAD and HF are independent of confounders, but they may slightly influence celiac disease and acute appendicitis. In addition, neither the single statistical effect nor the integrated statistics after meta-analysis found that colorectal cancer and irritable bowel syndrome contribute to CAD and HF, but after MVMR adjustment, a causal relationship was found, indicating that smoking and obesity may lead to their occurrence, which is in line with clinical and epidemiological evidence (52, 53).

This work has several strengths. First is the study design based on the MR method, which integrally analyzed the causal effects across 22 GDs on CAD and HF. Second, the dependability derives from applying the summary level data of the enormous GWAS datasets and subsequently following a meta-analysis and a bi-directional MR analysis to elucidate the anastrophic causation. We also conducted an MVMR analysis to mitigate horizontal pleiotropy caused by other underlying factors.

Nonetheless, the work has several limitations: 1. Our study only employed data from European ancestry, which is likely slanted by the population structure bias. 2. Because the number of SNPs associated with exposure as IVs did not satisfy the significant genome-wide because of stringent screening conditions, we relaxed the threshold for *P* value, a common approach to deal with too few SNPs. 3. We adjusted the effect estimates for multiple testing via Benjamini-Hochgerg, which was a relatively relaxing way. However, we repeated the analysis in different datasets, largely avoiding false positives. 4. There were certain horizontal pleiotropies in MR-Egger, implying that GDs-related SNPs result in CAD through other traits. Still, we conducted MVMR analysis adjusting for smoking and BMI and replicated analyses after removing outliers detected in MR-PRESSO. Besides, we assessed heterogeneity and horizontal pleiotropy primarily through Q-tests and Egger intercepts. Although this statistically removes heterogeneity and horizontal pleiotropy, there is not yet a complete guarantee that heterogeneity and horizontal pleiotropy do not exist in the clinical setting, which may be affected by differences in the GWAS datasets used, phenotype definitions, or disease classifications. Summary data MR relies on obtaining SNP‐trait associations from a GWAS, which usually integrates data from many studies. Therefore, we used Meta-analysis to combine results from separate epidemiological studies as the final causal associations, which reduces the heterogeneity to some extent. 5. Owing to all exposures defined as binary phenotypes, the causations may be predetermined by the hypothesis of exclusion restriction (54). However, GDs are not only diagnosed by continuous indicators but also by clinical symptoms and others, suggesting that we tried to uphold this assumption. In a word, we hope to resolve the deficiencies mentioned above in future studies with the emerging public availability of GWAS data in diverse regions worldwide and the demonstration of these causalities based on epidemiologic studies.

## 5 Conclusion

In summary, this MR study detected a series of GDs, of which genetic liability to GERD, celiac disease and duodenal ulcer are risk factors for CAD, and GERD and celiac disease probably increase the risk of HF. Besides, these results are independent of smoking and BMI after MVMR adjustment. Additionally, we also found a broad range of causal links of GDs on CAD and HF only in individual consortiums, whereas these causalities disappeared in summary statistics by the meta-analysis, but we cannot curtly rule out these weak associations. Our discoveries may shed light on the early recognition and appropriate management of GDs in patients with CAD and HF.

## Data Availability

The datasets presented in this study can be found in online repositories. The names of the repository/repositories and accession number(s) can be found in the article/**Supplementary Material**.

## References

[1] G. B. D. D. Collaborators. Global age-sex-specific fertility, mortality, healthy life expectancy (HALE), and population estimates in 204 countries and territories 1950-2019: a comprehensive demographic analysis for the Global Burden of Disease Study 2019. Lancet. (2020) 396:1160–203. doi: 10.1016/S0140-6736(20)30977-6 PMC7566045 33069325

[2] Amin AM. Metabolomics applications in coronary artery disease personalized medicine. Adv Clin Chem. (2021) 102:233–70. doi: 10.1016/bs.acc.2020.08.003 34044911

[3] Lesnefsky EJ Moghaddas S Tandler B Kerner J Hoppel CL. Mitochondrial dysfunction in cardiac disease: ischemia–reperfusion, aging, and heart failure. J Mol Cell Cardiol. (2001) 33:1065–89. doi: 10.1006/jmcc.2001.1378 11444914

[4] Dhingra R Vasan RS. Biomarkers in cardiovascular disease: Statistical assessment and section on key novel heart failure biomarkers. Trends Cardiovasc Med. (2017) 27:123–33. doi: 10.1016/j.tcm.2016.07.005 PMC5253084 27576060

[5] Piran S Liu P Morales A Hershberger RE. Where genome meets phenome: rationale for integrating genetic and protein biomarkers in the diagnosis and management of dilated cardiomyopathy and heart failure. J Am Coll Cardiol. (2012) 60:283–9. doi: 10.1016/j.jacc.2012.05.005 22813604

[6] Malakar AK Choudhury D Halder B Paul P Uddin A Chakraborty S. A.-O. A review on coronary artery disease, its risk factors, and therapeutics. (2019) 234:16812–23. doi: 10.1002/jcp.28350 30790284

[7] Tang WH Hazen SL. The contributory role of gut microbiota in cardiovascular disease. J Clin Invest. (2014) 124:4204–11. doi: 10.1172/JCI72331 PMC4215189 25271725

[8] Wahlström A Sayin SI Marschall HU Bäckhed F. Intestinal crosstalk between bile acids and microbiota and its impact on host metabolism. Cell Metab. (2016) 24:41–50. doi: 10.1016/j.cmet.2016.05.005 27320064

[9] Koh A De Vadder F Kovatcheva-Datchary P Bäckhed F. From dietary fiber to host physiology: short-chain fatty acids as key bacterial metabolites. Cell. (2016) 165:1332–45. doi: 10.1016/j.cell.2016.05.041 27259147

[10] Liu H Chen X Hu X Niu H Tian R Wang H. Alterations in the gut microbiome and metabolism with coronary artery disease severity. Microbiome. (2019) 7:68. doi: 10.1186/s40168-019-0683-9 31027508 PMC6486680

[11] Ubhi K Masliah E. Alzheimer's disease: recent advances and future perspectives. J Alzheimers Dis. (2013) 33:S185–194. doi: 10.3233/JAD-2012-129028 22810100

[12] Bisgaard TA-O Allin KA-O Keefer L Ananthakrishnan AN Jess TA-O. Depression and anxiety in inflammatory bowel disease: epidemiology, mechanisms and treatment. Nat Rev Gastroenterol Hepatol. (2022) 19:717–26. doi: 10.1038/s41575-022-00634-6 35732730

[13] Du YA-O Rayner CK Jones KL Talley NJ Horowitz MA-O. Gastrointestinal symptoms in diabetes: prevalence, assessment, pathogenesis, and management. Diabetes Care. (2018) 41:627–37. doi: 10.2337/dc17-1536 29463666

[14] Gesualdo M Scicchitano P Carbonara S Ricci G Principi M Ierardi E. The association between cardiac and gastrointestinal disorders: causal or casual link? J Cardiovasc Med (Hagerstown). (2016) 17:330–8. doi: 10.2459/JCM.0000000000000351 26702598

[15] Anstee QM Mantovani A Tilg H Targher GA-O. Risk of cardiomyopathy and cardiac arrhythmias in patients with nonalcoholic fatty liver disease. Nat Rev Gastroenterol Hepatol. (2018) 15:425–439. doi: 10.1038/s41575-018-0010-0 29713021

[16] Wiese S Hove JD Bendtsen F Møller S. Cirrhotic cardiomyopathy: pathogenesis and clinical relevance. Nat Rev Gastroenterol Hepatol. (2014) 11:177–86. doi: 10.1038/nrgastro.2013.210 24217347

[17] Kato H Ishii T Akimoto T Urita Y Sugimoto M. Prevalence of linked angina and gastroesophageal reflux disease in general practice. World J Gastroenterol. (2009) 15:1764–8. doi: 10.3748/wjg.15.1764 PMC2668783 19360921

[18] Kristensen SL Ahlehoff O Lindhardsen J Erichsen R Jensen GV Torp-Pedersen C. Disease activity in inflammatory bowel disease is associated with increased risk of myocardial infarction, stroke and cardiovascular death–a Danish nationwide cohort study. PLoS One. (2013) 8:e56944. doi: 10.1371/journal.pone.0056944 23457642 PMC3574079

[19] West J Logan RF Card TR Smith C Hubbard R. Risk of vascular disease in adults with diagnosed coeliac disease: a population-based study. Aliment Pharmacol Ther. (2004) 20:73–9. doi: 10.1111/j.1365-2036.2004.02008.x 15225173

[20] Lawlor DA Harbord RM Sterne JA Timpson N Davey Smith G. Mendelian randomization: using genes as instruments for making causal inferences in epidemiology. Stat Med. (2008) 27:1133–63. doi: 10.1002/sim.3034 17886233

[21] Davies NM Holmes MV Davey Smith G. Reading Mendelian randomisation studies: a guide, glossary, and checklist for clinicians. BMJ Med. (2018) 12:k601. doi: 10.1136/bmj.k601 PMC6041728 30002074

[22] Pingault JB O'Reilly PF Schoeler T Ploubidis GB Rijsdijk F Dudbridge F. Using genetic data to strengthen causal inference in observational research. Nat Rev Genet. (2018) 19:566–80. doi: 10.1038/s41576-018-0020-3 29872216

[23] Sun X Chen L Zheng LA-O. A Mendelian randomization study to assess the genetic liability of gastroesophageal reflux disease for cardiovascular diseases and risk factors. Hum Mol Genet. (2022) 31:4275–85. doi: 10.1093/hmg/ddac162 35861629

[24] Huang J. Assessment of the causal association between celiac disease and cardiovascular diseases. Front Cardiovasc Med. (2022) 9:1017209. doi: 10.3389/fcvm.2022.1017209 36386312 PMC9644835

[25] Qiu X Hou C Yang Z Wang Q Li L. Inflammatory bowel disease and risk of coronary heart disease: A Mendelian randomization study. Wien Klin Wochenschr. (2022) 134:779–87. doi: 10.1007/s00508-022-02095-y 36239805

[26] Lala A Desai AS. The role of coronary artery disease in heart failure. Heart Fail Clin. (2014) 10:353–65. doi: 10.1016/j.hfc.2013.10.002 24656111

[27] Sudlow C Gallacher J Allen N Beral V Burton P Danesh J. UK biobank: an open access resource for identifying the causes of a wide range of complex diseases of middle and old age. PLoS Med. (2015) 12:e1001779. doi: 10.1371/journal.pmed.1001779 25826379 PMC4380465

[28] Machiela MJ Chanock SJ. LDlink: a web-based application for exploring population-specific haplotype structure and linking correlated alleles of possible functional variants. Bioinformatics. (2015) 31:3555–7. doi: 10.1093/bioinformatics/btv402 PMC4626747 26139635

[29] Nikpay M Goel A Won HH Hall LM Willenborg C Kanoni S. A comprehensive 1,000 Genomes-based genome-wide association meta-analysis of coronary artery disease. Nat Genet. (2015) 47:1121–30. doi: 10.1038/ng.3396 PMC4589895 26343387

[30] Shah SA-O Henry AA-O Roselli CA-O Lin HA-O Sveinbjörnsson G Fatemifar G. Genome-wide association and Mendelian randomisation analysis provide insights into the pathogenesis of heart failure. Nat Commun. (2020) 11:163. doi: 10.1038/s41467-019-13690-5 31919418 PMC6952380

[31] Krawczyk P Święcicki Ł. ICD-11 vs. ICD-10 - a review of updates and novelties introduced in the latest version of the WHO International Classification of Diseases. Psychiatr Pol. (2020) 54:7–20. doi: 10.12740/PP/103876 32447353

[32] Bowden J Del Greco MF Minelli C Davey Smith G Sheehan N Thompson J. A framework for the investigation of pleiotropy in two-sample summary data Mendelian randomization. Stat Med. (2017) 36:1783–802. doi: 10.1002/sim.v36.11 PMC5434863 28114746

[33] Bowden J Davey Smith G Haycock PC Burgess S. Consistent estimation in mendelian randomization with some invalid instruments using a weighted median estimator. Genet Epidemiol. (2016) 40:304–14. doi: 10.1002/gepi.2016.40.issue-4 PMC4849733 27061298

[34] Bowden J Davey Smith G Burgess S. Mendelian randomization with invalid instruments: effect estimation and bias detection through Egger regression. Int J Epidemiol. (2015) 44:512–25. doi: 10.1093/ije/dyv080 PMC4469799 26050253

[35] Verbanck M Chen CA-O Neale BA-O Do RA-O. Detection of widespread horizontal pleiotropy in causal relationships inferred from Mendelian randomization between complex traits and diseases. Nat Genet. (2018) 50:693–8. doi: 10.1038/s41588-018-0099-7 PMC6083837 29686387

[36] Verbanck M Chen CA-O Neale BA-O Do RA-O. Detection of widespread horizontal pleiotropy in causal relationships inferred from Mendelian randomization between complex traits and diseases. Nat Genet. 50:693–8. doi: 10.1038/s41588-018-0099-7 PMC6083837 29686387

[37] Borenstein M Hedges LV Higgins JP Rothstein HR. A basic introduction to fixed-effect and random-effects models for meta-analysis. Res Synth Methods. (2010) 1:97–111. doi: 10.1002/jrsm.12 26061376

[38] Hemani GA-O Tilling K Davey Smith GA-O. Orienting the causal relationship between imprecisely measured traits using GWAS summary data. PLoS Genet. (2017) 17:e1007081. doi: 10.1371/journal.pgen.1007081 PMC5711033 29149188

[39] Hemani GA-O Zheng JA-O Elsworth B Wade KA-O Haberland V Baird DA-O. The MR-Base platform supports systematic causal inference across the human phenome. Elife. (2018) 30:e34408. doi: 10.7554/eLife.34408 PMC5976434 29846171

[40] Trent JC Patel SS Zhang J Araujo DM Plana JC Lenihan DJ. Rare incidence of congestive heart failure in gastrointestinal stromal tumor and other sarcoma patients receiving imatinib mesylate. Cancer. (2010) 116:184–92. doi: 10.1002/cncr.24683 PMC4306337 19885836

[41] O'Ryan M Prado V Pickering LK. A millennium update on pediatric diarrheal illness in the developing world. Semin Pediatr Infect Dis. (2005) 16:125–36. doi: 10.1053/j.spid.2005.12.008 15825143

[42] Schlech WF. Epidemiology and clinical manifestations of listeria monocytogenes infection. Microbiol Spectr. (2019) 7. doi: 10.1128/microbiolspec.GPP3-0014-2018 PMC11026082 31837132

[43] Hunt R Armstrong D Katelaris P Afihene M Bane A Bhatia S. World gastroenterology organisation global guidelines: GERD global perspective on gastroesophageal reflux disease. J Clin Gastroenterol. (2017) 51:467–78. doi: 10.1097/MCG.0000000000000854 28591069

[44] Dobrzycki S Baniukiewicz A Korecki J Bachórzewska-Gajewska H Prokopczuk P Musial WJ. Does gastro-esophageal reflux provoke the myocardial ischemia in patients with CAD? Int J Cardiol. (2005) 15:67–72. doi: 10.1016/j.ijcard.2004.10.018 16137512

[45] Kristensen SL Ahlehoff O Lindhardsen J Erichsen R Lamberts M Khalid U. Inflammatory bowel disease is associated with an increased risk of hospitalization for heart failure: a Danish Nationwide Cohort study. Circ Heart Fail. (2014) 7:717–22. doi: 10.1161/CIRCHEARTFAILURE.114.001152 25052190

[46] Ludvigsson JF James S Askling J Stenestrand U Ingelsson E. Nationwide cohort study of risk of ischemic heart disease in patients with celiac disease. Circulation. (2011) 123:483–90. doi: 10.1161/CIRCULATIONAHA.110.965624 21262996

[47] Danese S Papa A Saibeni S Repici A Malesci A Vecchi M. Inflammation and coagulation in inflammatory bowel disease: The clot thickens. Am J Gastroenterol. (2007) 102:174–86. doi: 10.1111/j.1572-0241.2006.00943.x 17100967

[48] Nema H Kato M Katsurada T Nozaki Y Yotsukura A Yoshida I. Investigation of gastric and duodenal mucosal defects caused by low-dose aspirin in patients with ischemic heart disease. J Clin Gastroenterol. (2009) 43:130–32. doi: 10.1097/MCG.0b013e3181580e8a 18779739

[49] Conroy M Allen N Lacey B Soilleux E Littlejohns T. Association between coeliac disease and cardiovascular disease: prospective analysis of UK Biobank data. BMJ Med. (2023) 2:e000371. doi: 10.1136/bmjmed-2022-000371 PMC9951384 36936262

[50] Mostofsky E Chahal HS Mukamal KJ Rimm EB Mittleman MA. Alcohol and immediate risk of cardiovascular events: A systematic review and dose-response meta-analysis. Circulation. (2016) 133:979–87. doi: 10.1161/CIRCULATIONAHA.115.019743 PMC4783255 26936862

[51] Ambrose JA Barua RS. The pathophysiology of cigarette smoking and cardiovascular disease: an update. J Am Coll Cardiol. (2004) 43:1731–7. doi: 10.1016/j.jacc.2003.12.047 15145091

[52] Yang P Zhou Y Chen B Wan HW Jia GQ Bai HL. Overweight, obesity and gastric cancer risk: results from a meta-analysis of cohort studies. Eur J Cancer. (2009) 45:2867–73. doi: 10.1016/j.ejca.2009.04.019 19427197

[53] Rota MA-O Possenti IA-O Valsassina V Santucci CA-O Bagnardi VA-O. Dose-response association between cigarette smoking and gastric cancer risk: a systematic review and meta-analysis. Gastric Cancer. (2024) 27:197–209. doi: 10.1007/s10120-023-01459-1 38231449

[54] Burgess S Labrecque JA. Mendelian randomization with a binary exposure variable: interpretation and presentation of causal estimates. Eur J Epidemiol. (2018) 33:947–52. doi: 10.1007/s10654-018-0424-6 PMC6153517 30039250

