# Supplementary material for: Causal association between gastrointestinal diseases and coronary artery disease: a bidirectional Mendelian randomization study
Source: Front Endocrinol (Lausanne). 2024 Oct 15;15:1458196. doi: 10.3389/fendo.2024.1458196 (PMC11518705; doi:10.3389/fendo.2024.1458196)
Supplement: Supplementary file 1 [file DataSheet1.docx]

**Additional File 2**

**Causal association between gastrointestinal diseases and coronary artery disease: a bidirectional Mendelian randomization study**

Zhuoxi Wang Jifang Ban Yabin Zhou Rui Qie*

Corresponding to Rui Qie, Preventive Treatment Center, First Affiliated Hospital of Heilongjiang University of Chinese Medicine, 26 Heping Road, Xiangfang District, Haerbin, Heilongjiang, China. Electronic address: [wangzhuoxi0919@163.com](mailto:wangzhuoxi0919@163.com)


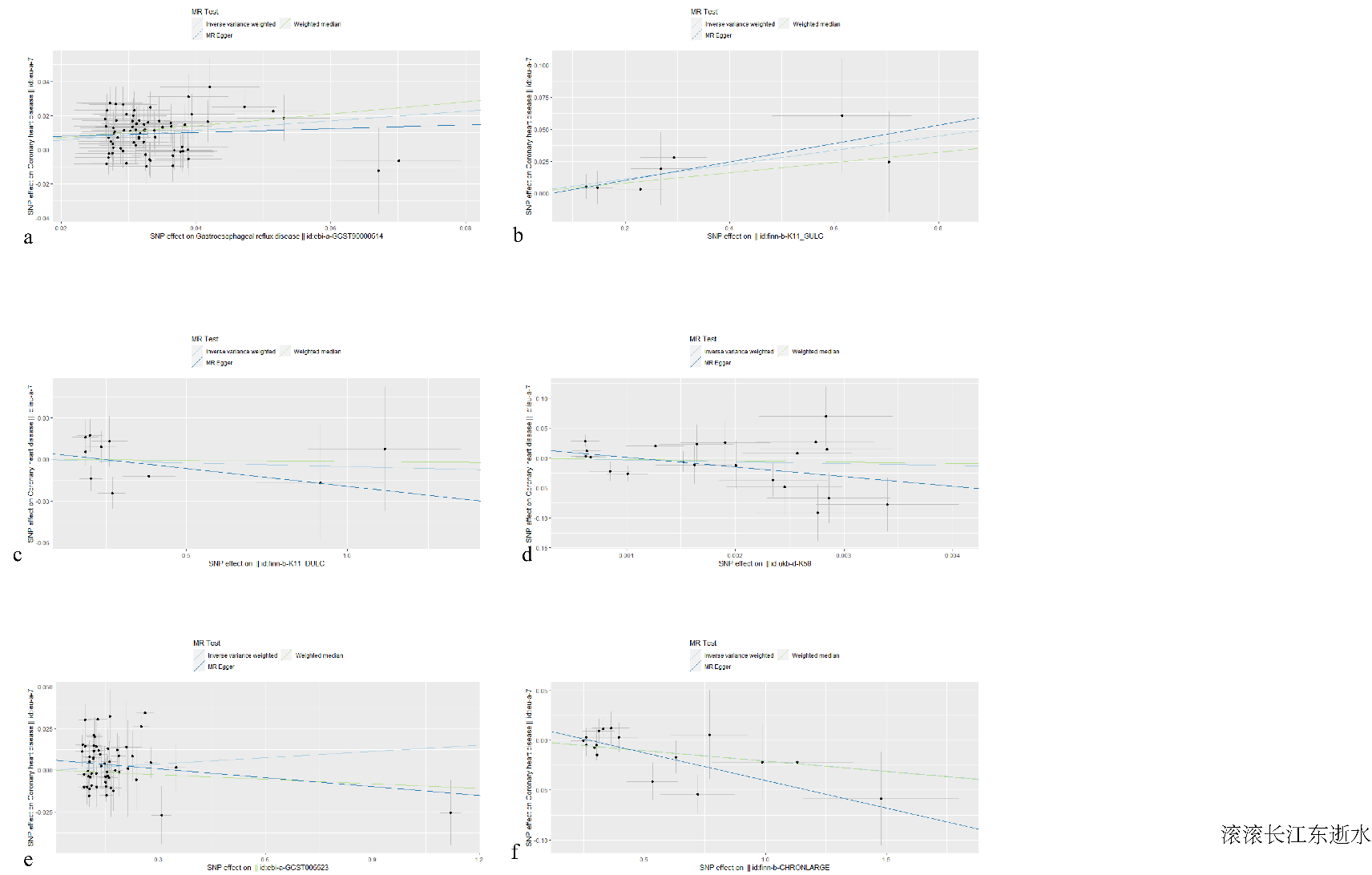


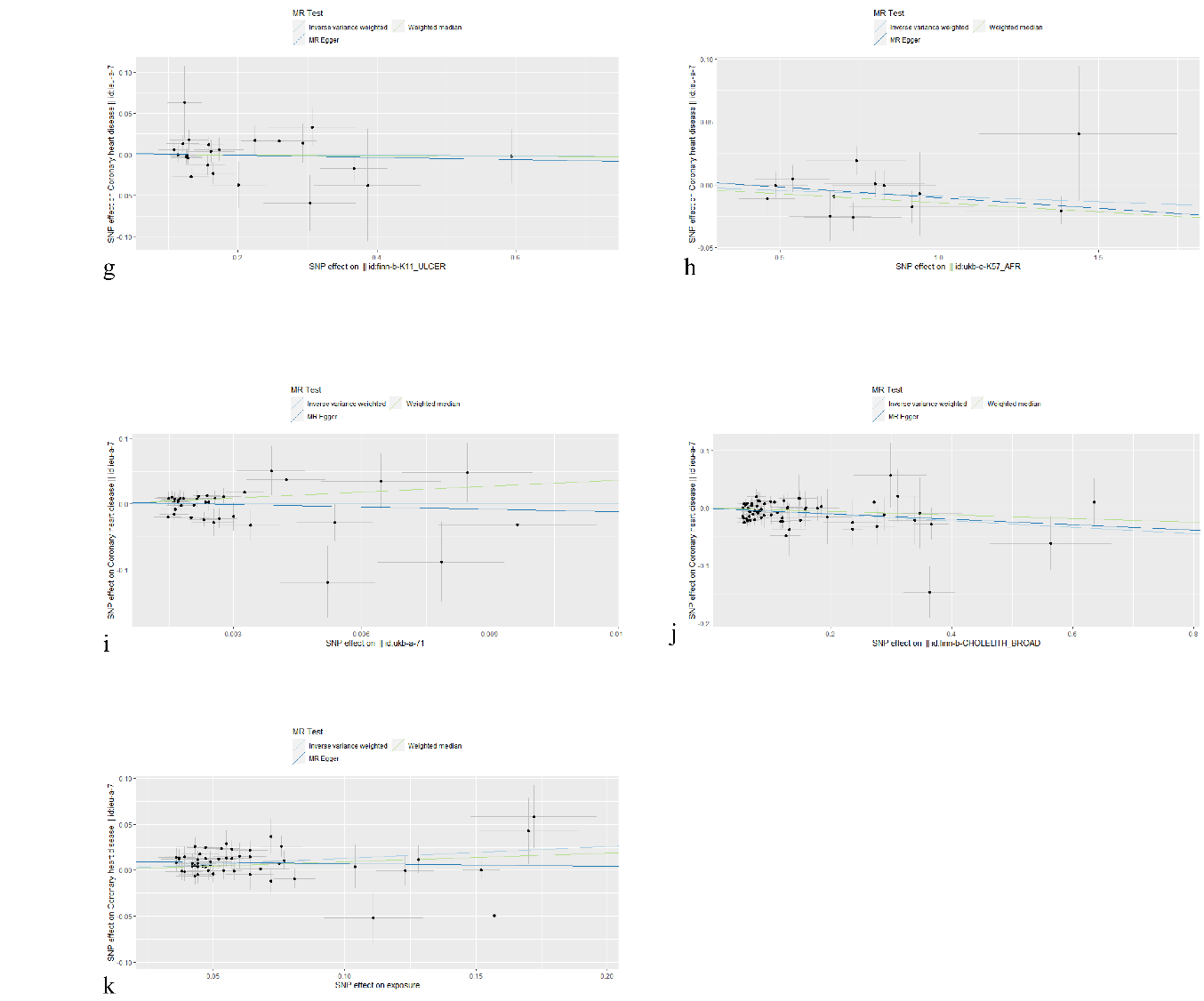


**Figure S1. Scatter plot of SNPs associated with gastrointestinal diseases and their risk of CAD in the CARDIoGRAMplusC4D consortium.** **(a)** Gastroesophageal reflux disease. **(b)** Gastric ulcer. **(c)** Duodenal ulcer. **(d)** Irritable bowel syndrome. **(e)** Celiac disease. **(f)** Crohn's disease. **(g)** Ulcerative colitis. **(h)** Diverticular disease. **(i)** Cholelithiasis. **(j)** Cholelithiasis with cholecystitis. **(k)** Non-alcoholic fatty liver disease.


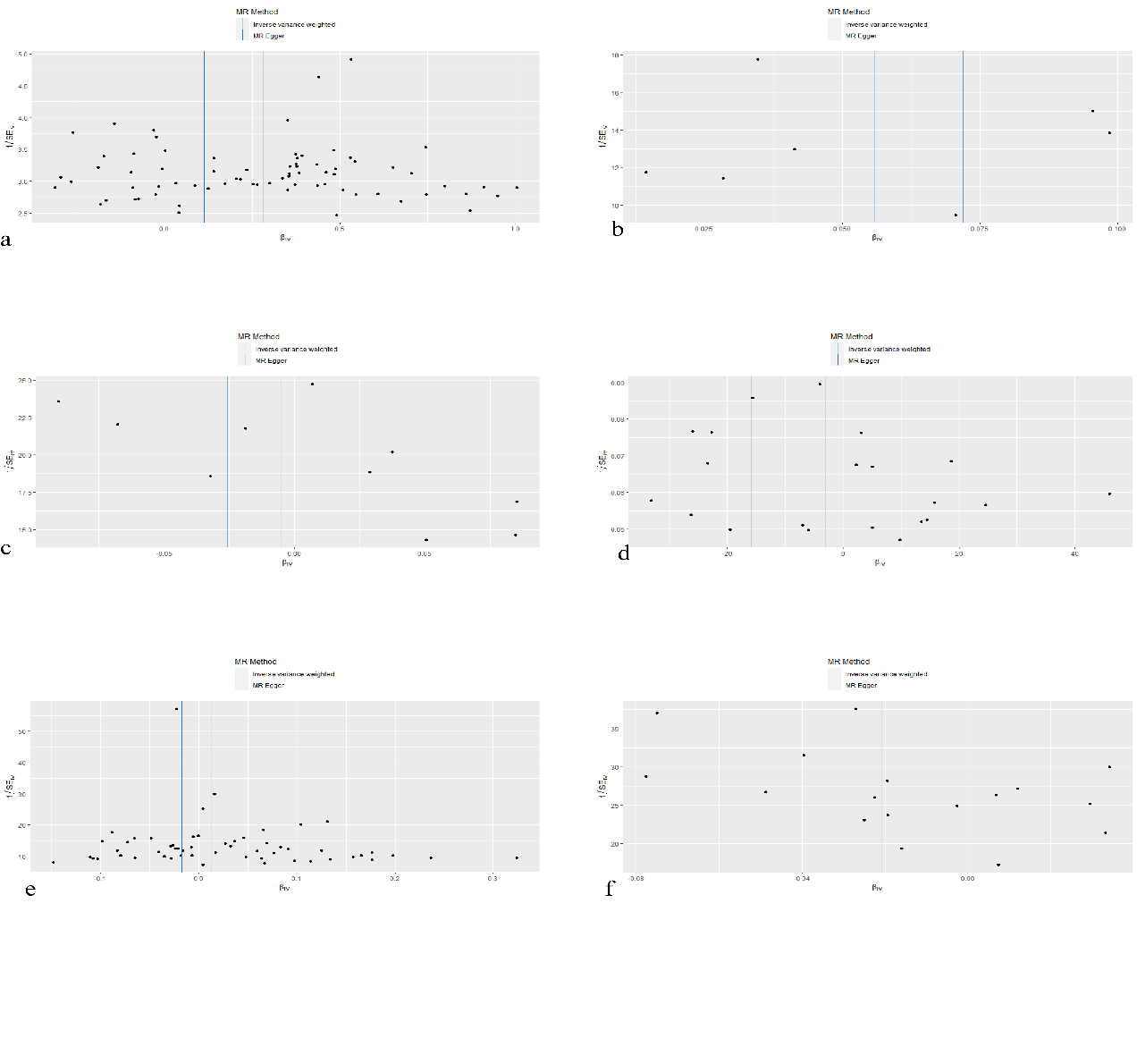


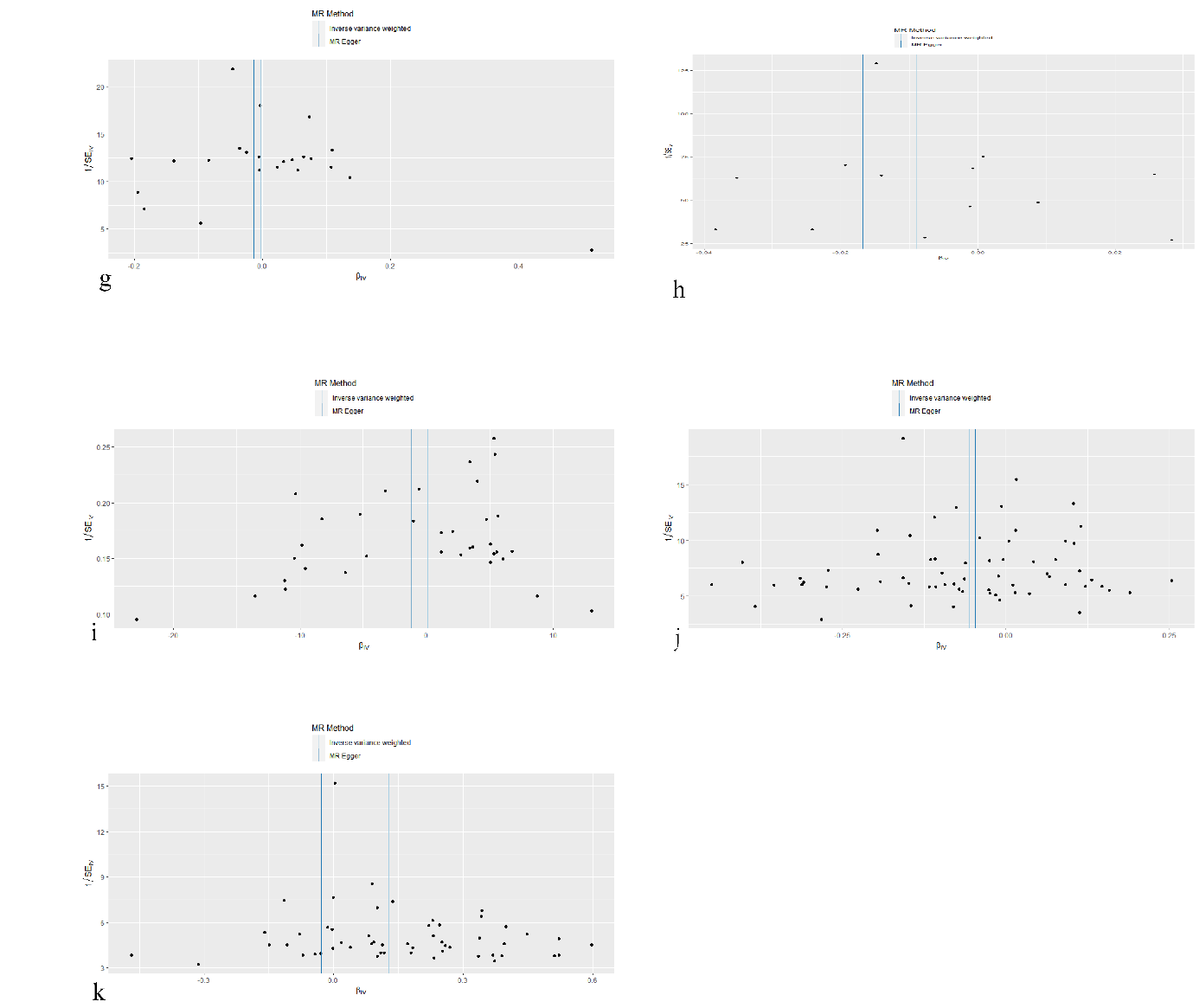


**Figure S2. Funnel plot of SNPs associated with gastrointestinal diseases and their risk of CAD in the CARDIoGRAMplusC4D consortium. (a)** Gastroesophageal reflux disease. **(b)** Gastric ulcer. **(c)** Duodenal ulcer. **(d)** Irritable bowel syndrome. **(e)** Celiac disease. **(f)** Crohn's disease. **(g)** Ulcerative colitis. **(h)** Diverticular disease. **(i)** Cholelithiasis. **(j)** Cholelithiasis with cholecystitis. **(k)** Non-alcoholic fatty liver disease.


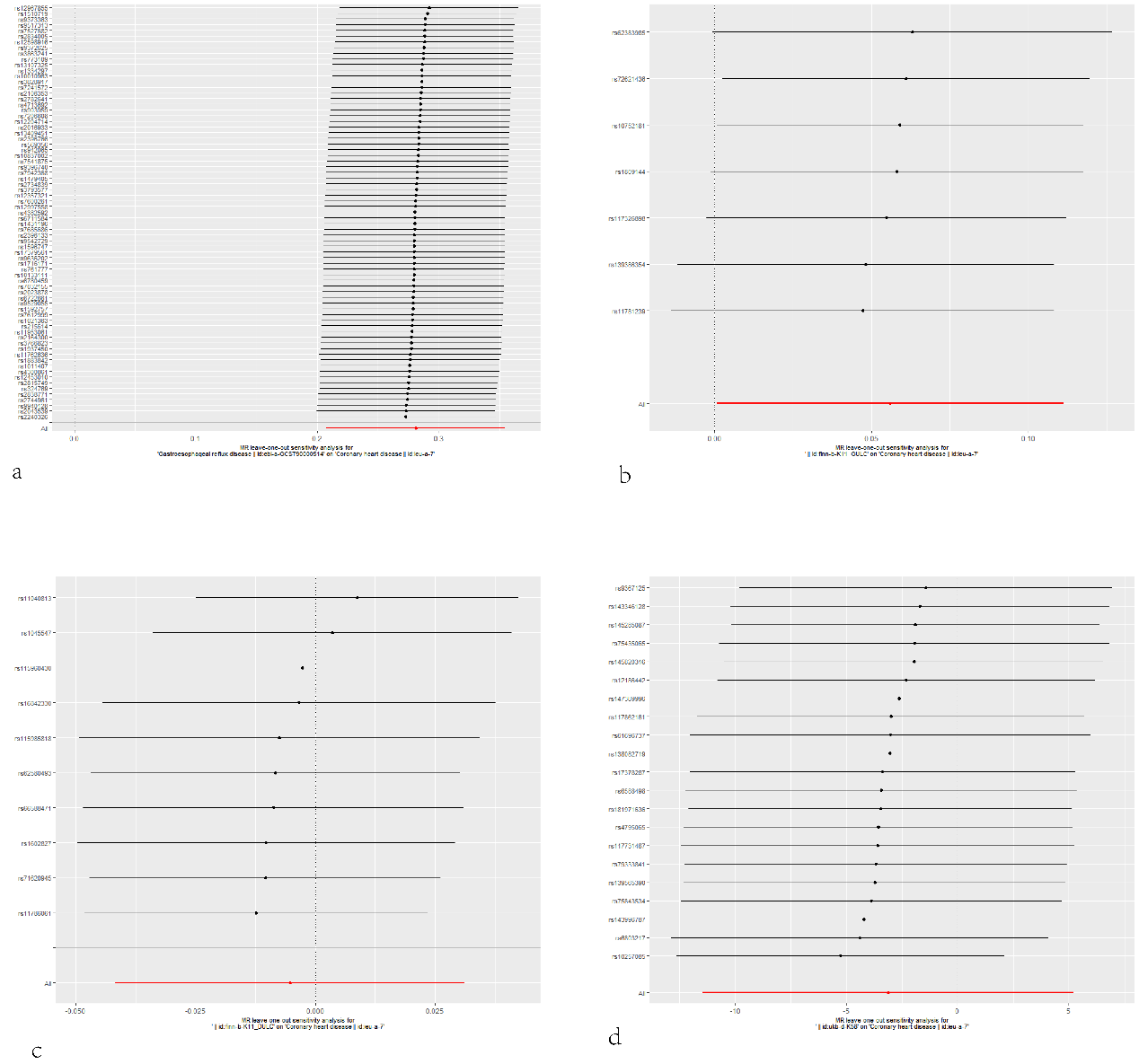


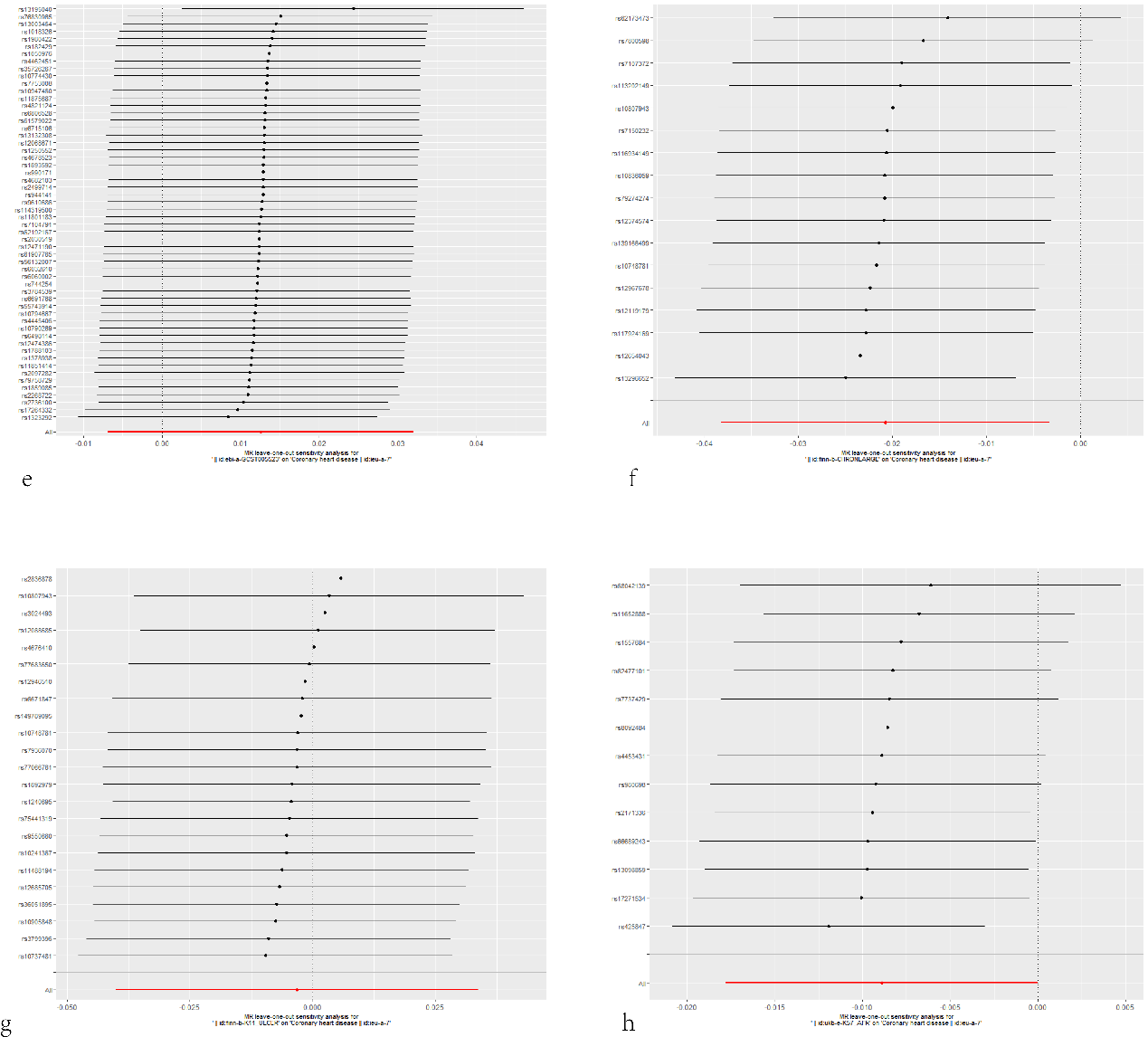


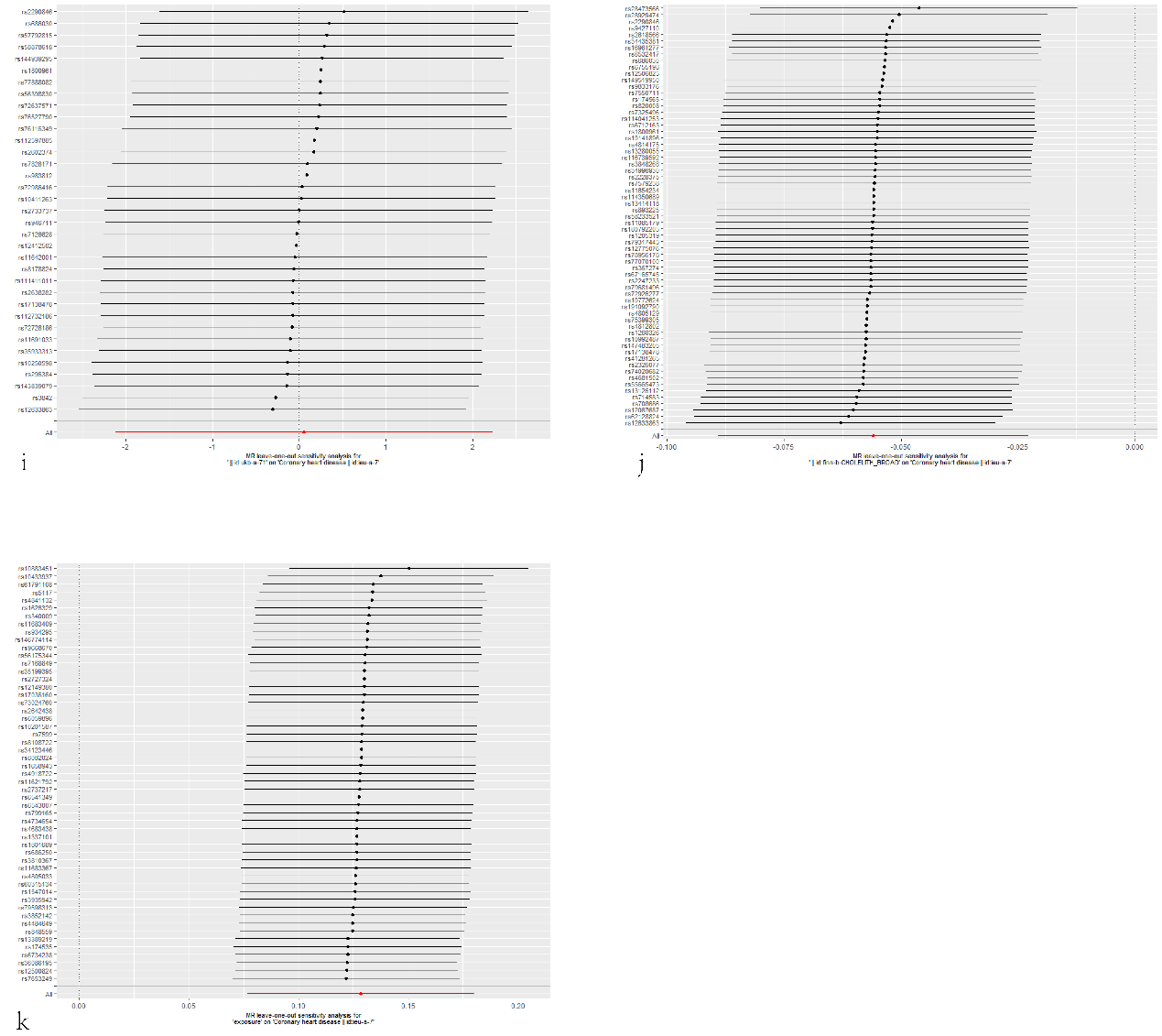


**Figure S3. Leave-one-out analysis of SNPs associated with gastrointestinal diseases and their risk of CAD in the CARDIoGRAMplusC4D consortium.** **(a)** Gastroesophageal reflux disease. **(b)** Gastric ulcer. **(c)** Duodenal ulcer. **(d)** Irritable bowel syndrome. **(e)** Celiac disease. **(f)** Crohn's disease. **(g)** Ulcerative colitis. **(h)** Diverticular disease. **(i)** Cholelithiasis. **(j)** Cholelithiasis with cholecystitis. **(k)** Non-alcoholic fatty liver disease.


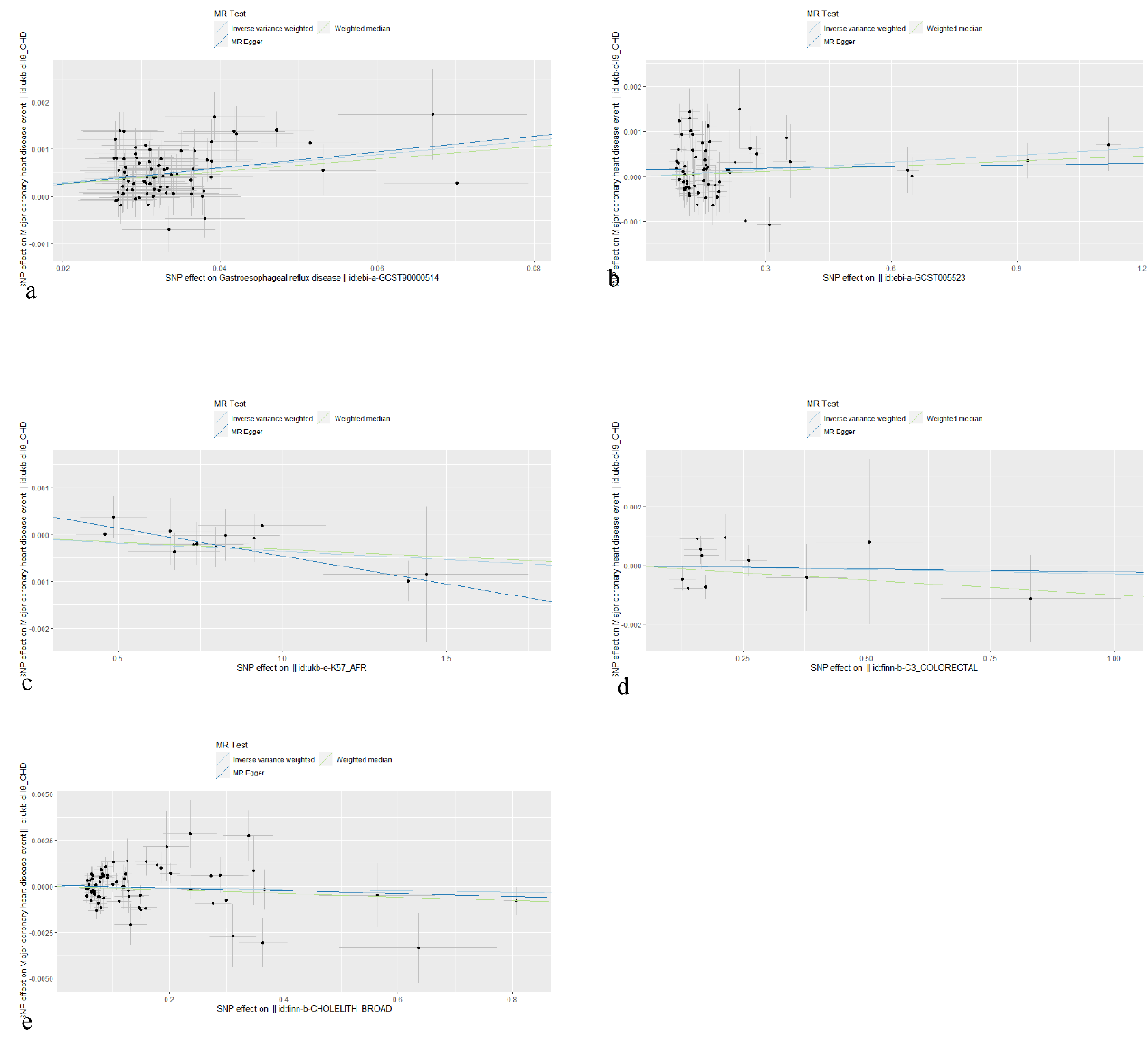


**Figure S4. Scatter plot of SNPs associated with gastrointestinal diseases and their risk of CAD in UK Biobank. (a)** Gastroesophageal reflux disease. **(b)** Celiac disease. **(c)** Diverticular disease. **(d)** Colorectal cancer. **(e)** Cholelithiasis with cholecystitis.


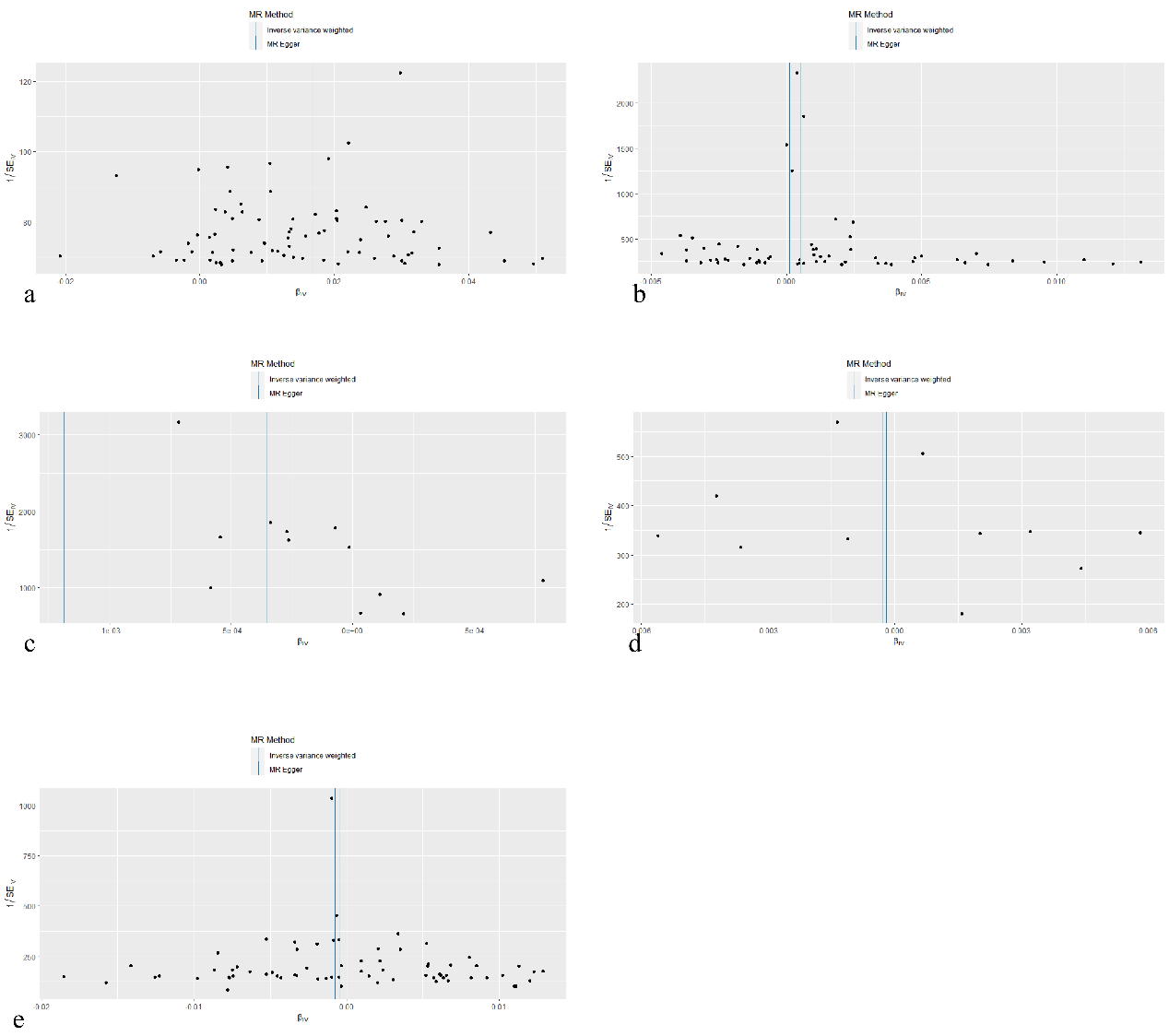


**Figure S5. Funnel plot of SNPs associated with gastrointestinal diseases and their risk of CAD in UK Biobank.** **(a)** Gastroesophageal reflux disease. **(b)** Celiac disease. **(c)** Diverticular disease. **(d)** Colorectal cancer. **(e)** Cholelithiasis with cholecystitis.


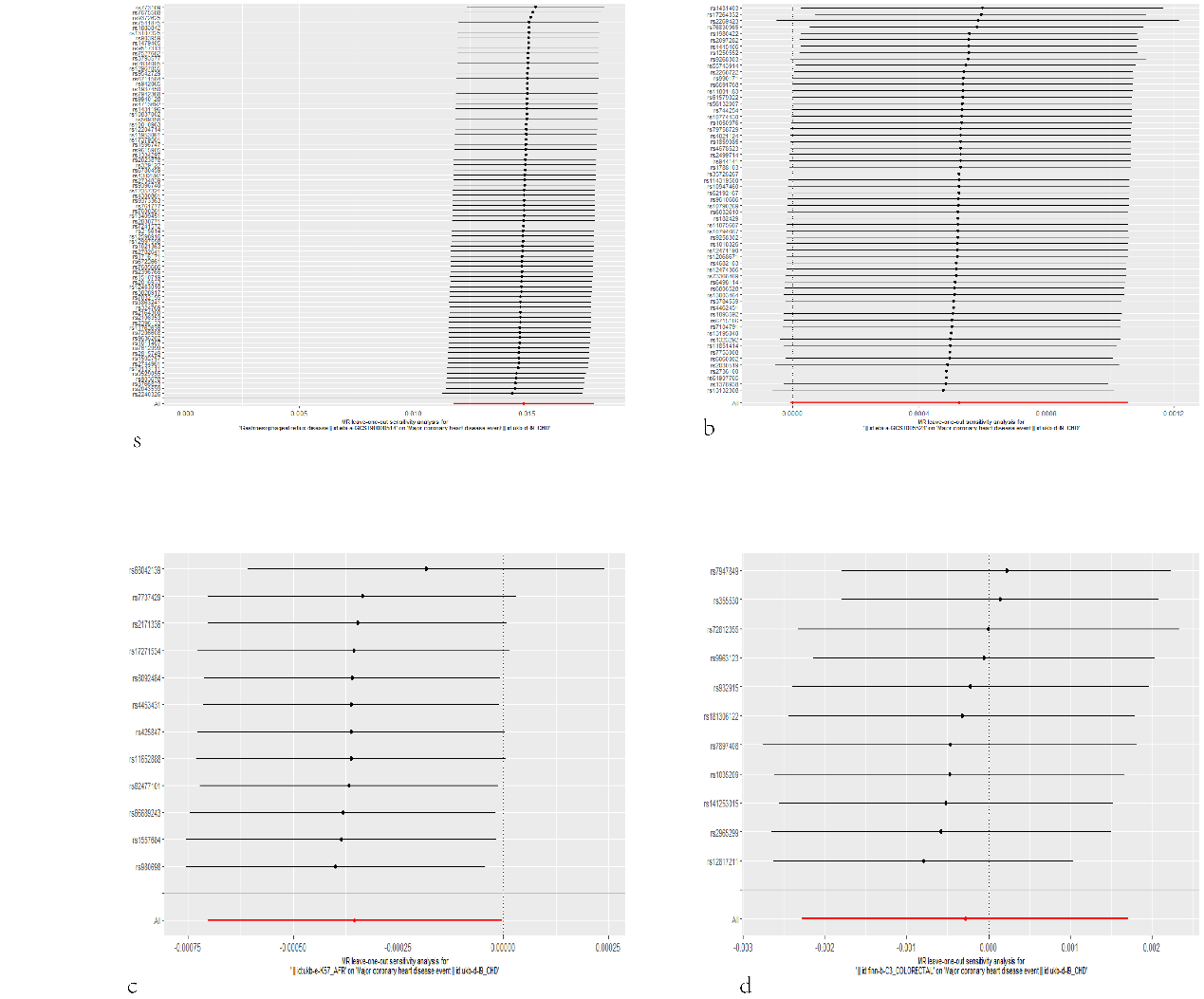


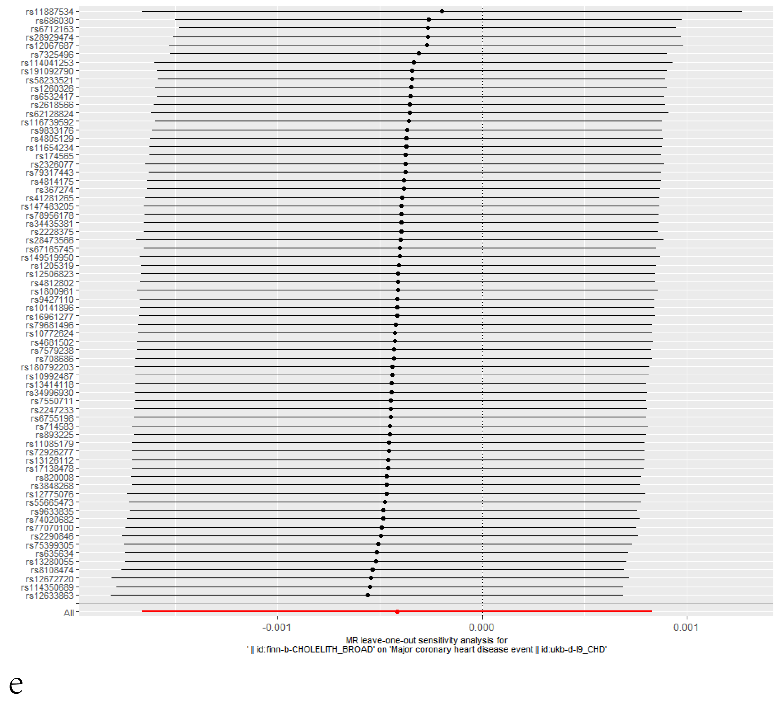


**Figure S6. Leave-one-out analysis of SNPs associated with gastrointestinal diseases and their risk of CAD in UK Biobank.** **(a)** Gastroesophageal reflux disease. **(b)** Celiac disease. **(c)** Diverticular disease. **(d)** Colorectal cancer. **(e)** Cholelithiasis with cholecystitis.


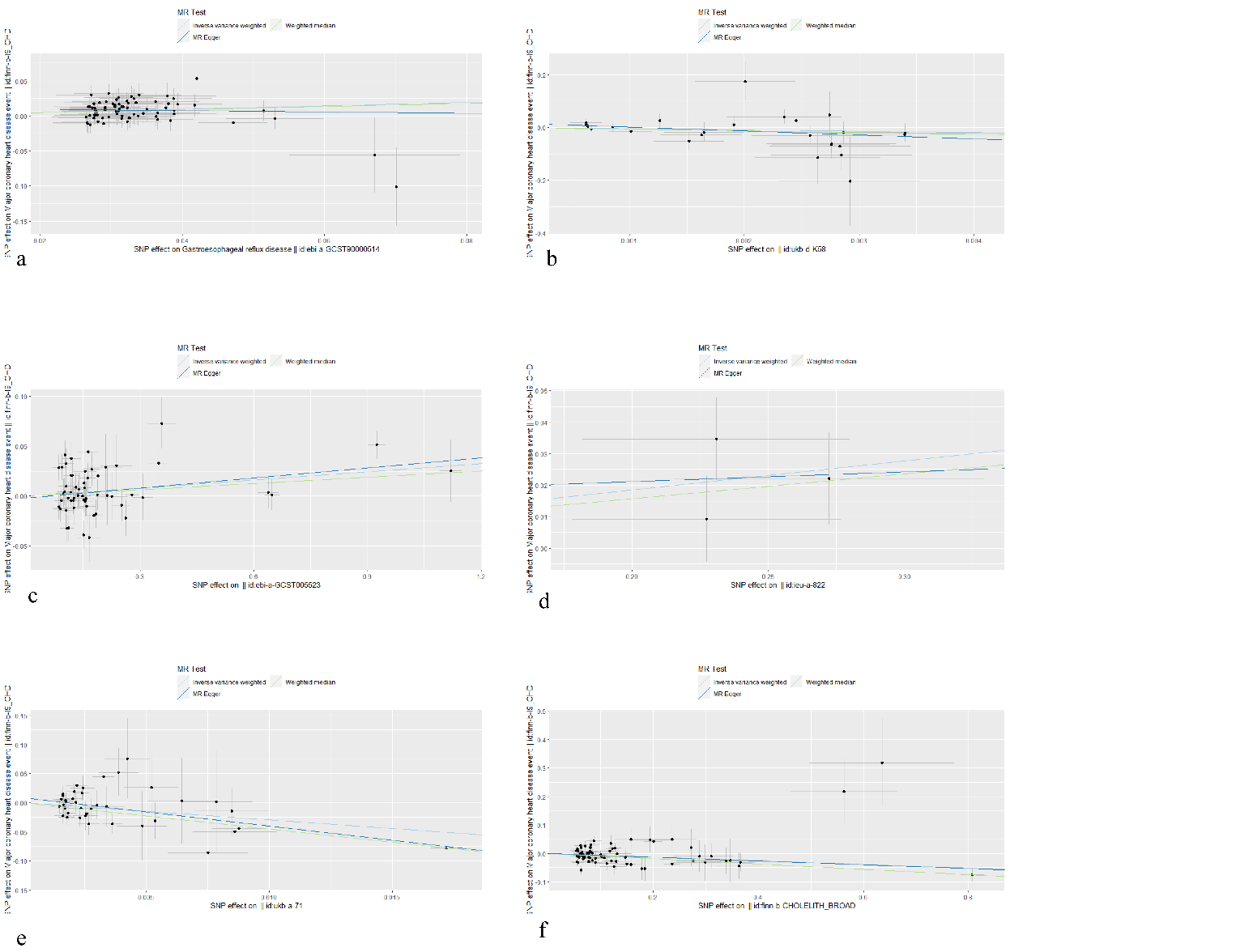


**Figure S7. Scatter plot of SNPs associated with gastrointestinal diseases and their risk of CAD in FinnGen.** **(a)** Gastroesophageal reflux disease. **(b)** Irritable bowel syndrome. **(c)** Celiac disease. **(d)** Pancreatic cancer. **(e)** Cholelithiasis. **(f)** Cholelithiasis with cholecystitis.


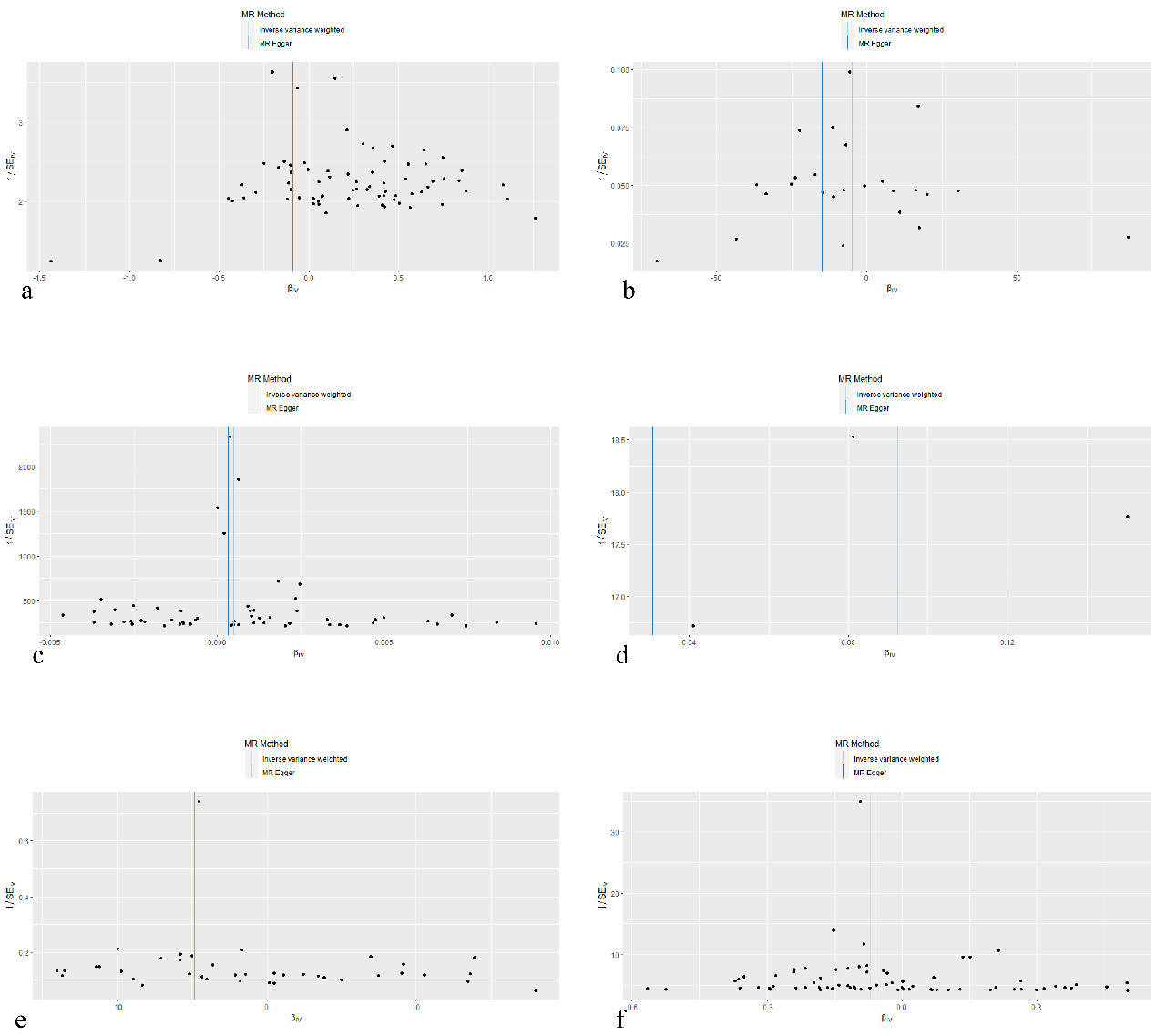


**Figure S8. Funnel plot of SNPs associated with gastrointestinal diseases and their risk of CAD in FinnGen.** **(a)** Gastroesophageal reflux disease. **(b)** Irritable bowel syndrome. **(c)** Celiac disease. **(d)** Pancreatic cancer. **(e)** Cholelithiasis. **(f)** Cholelithiasis with cholecystitis.


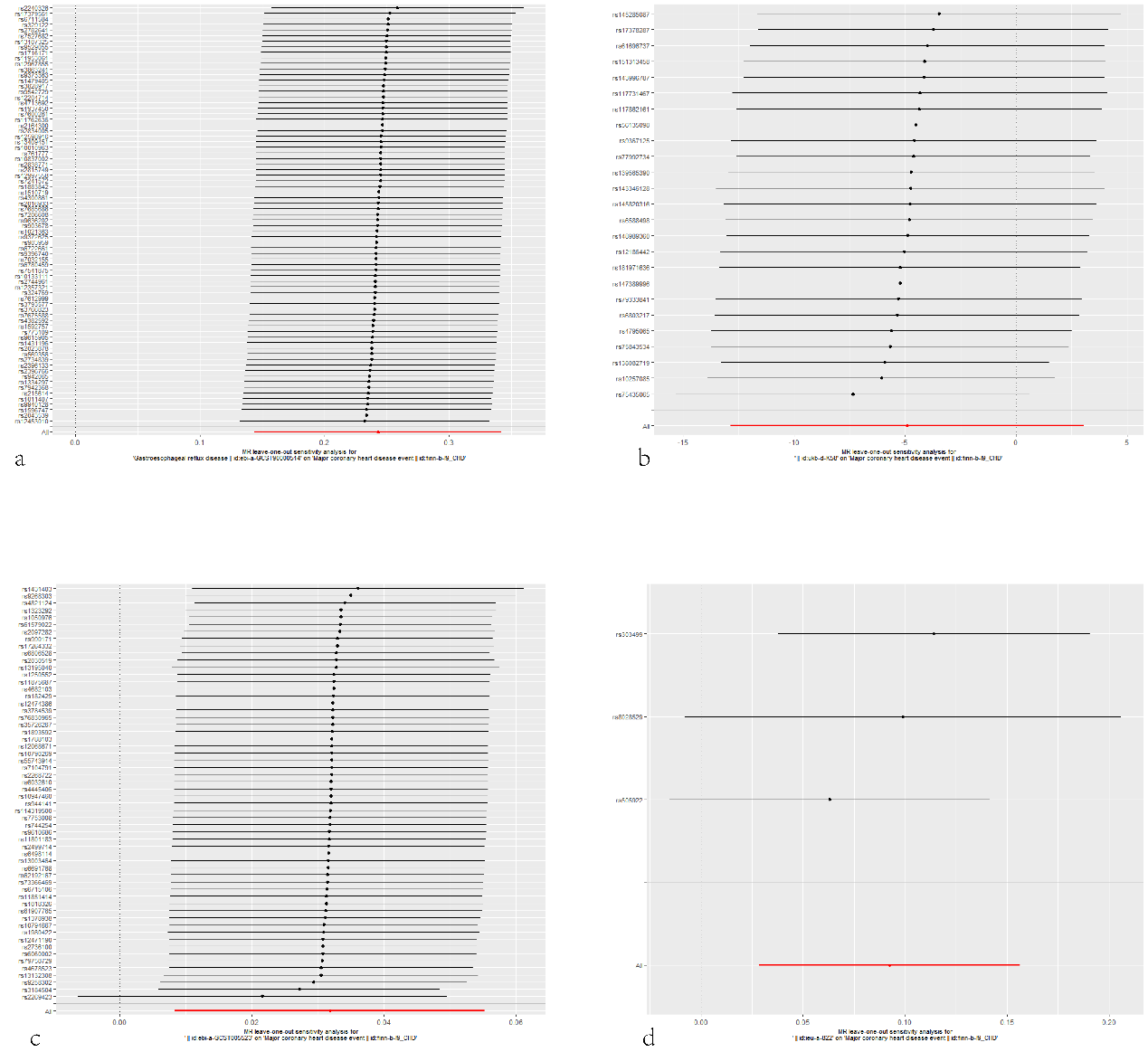


**Figure S9. Leave-one-out of SNPs associated with gastrointestinal diseases and their risk of CAD in FinnGen. (a)** Gastroesophageal reflux disease. **(b)** Irritable bowel syndrome. **(c)** Celiac disease. **(d)** Pancreatic cancer. **(e)** Cholelithiasis. **(f)** Cholelithiasis with cholecystitis.


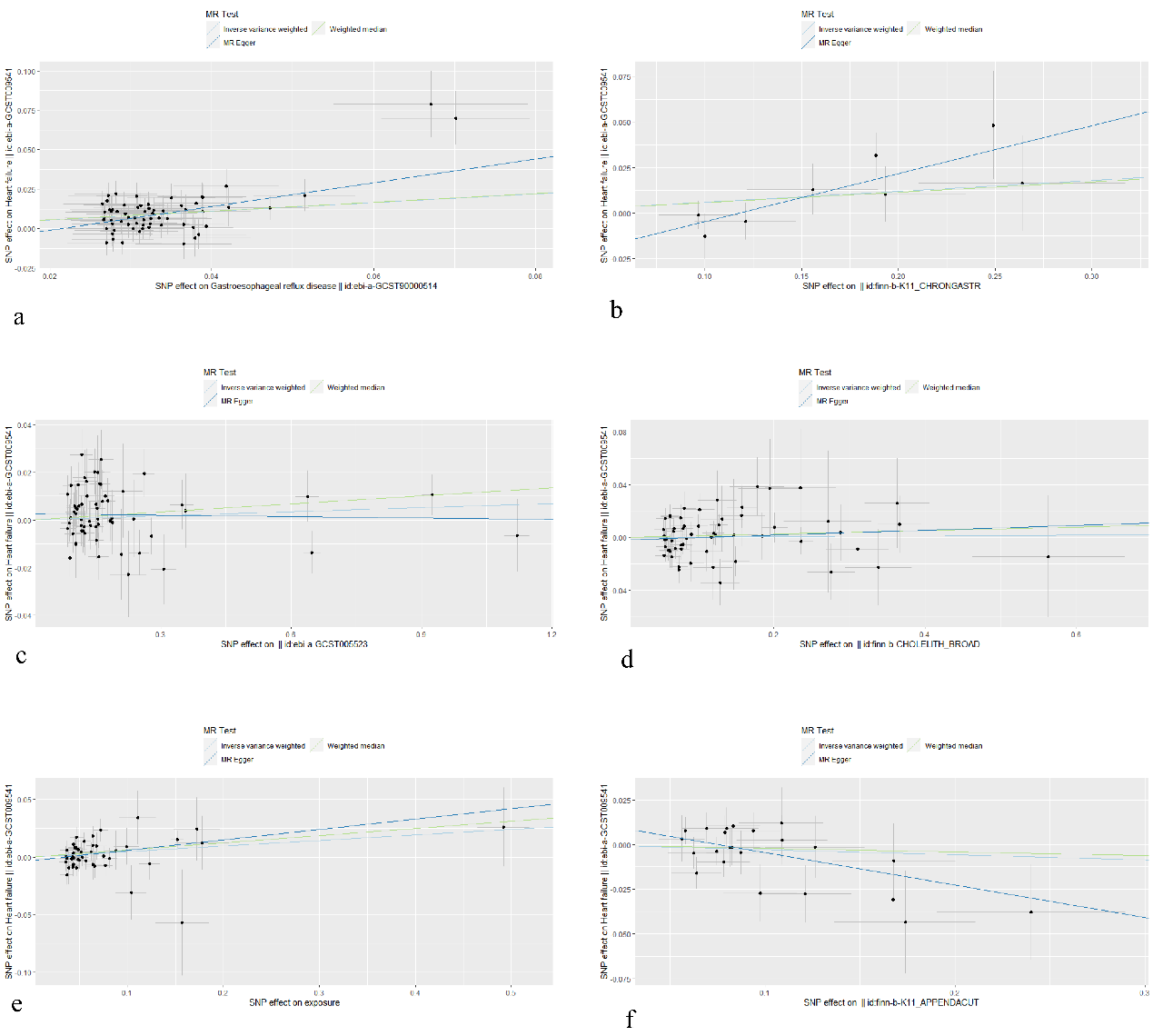


**Figure S10. Scatter plot of SNPs associated with gastrointestinal diseases and their risk of HF in the HERMES consortium. (a)** Gastroesophageal reflux disease. **(b)** Chronic gastritis. **(c)** Celiac disease. **(d)** Cholelithiasis with cholecystitis. **(e)** Non-alcoholic fatty liver disease. **(f)** Acute appendicitis.


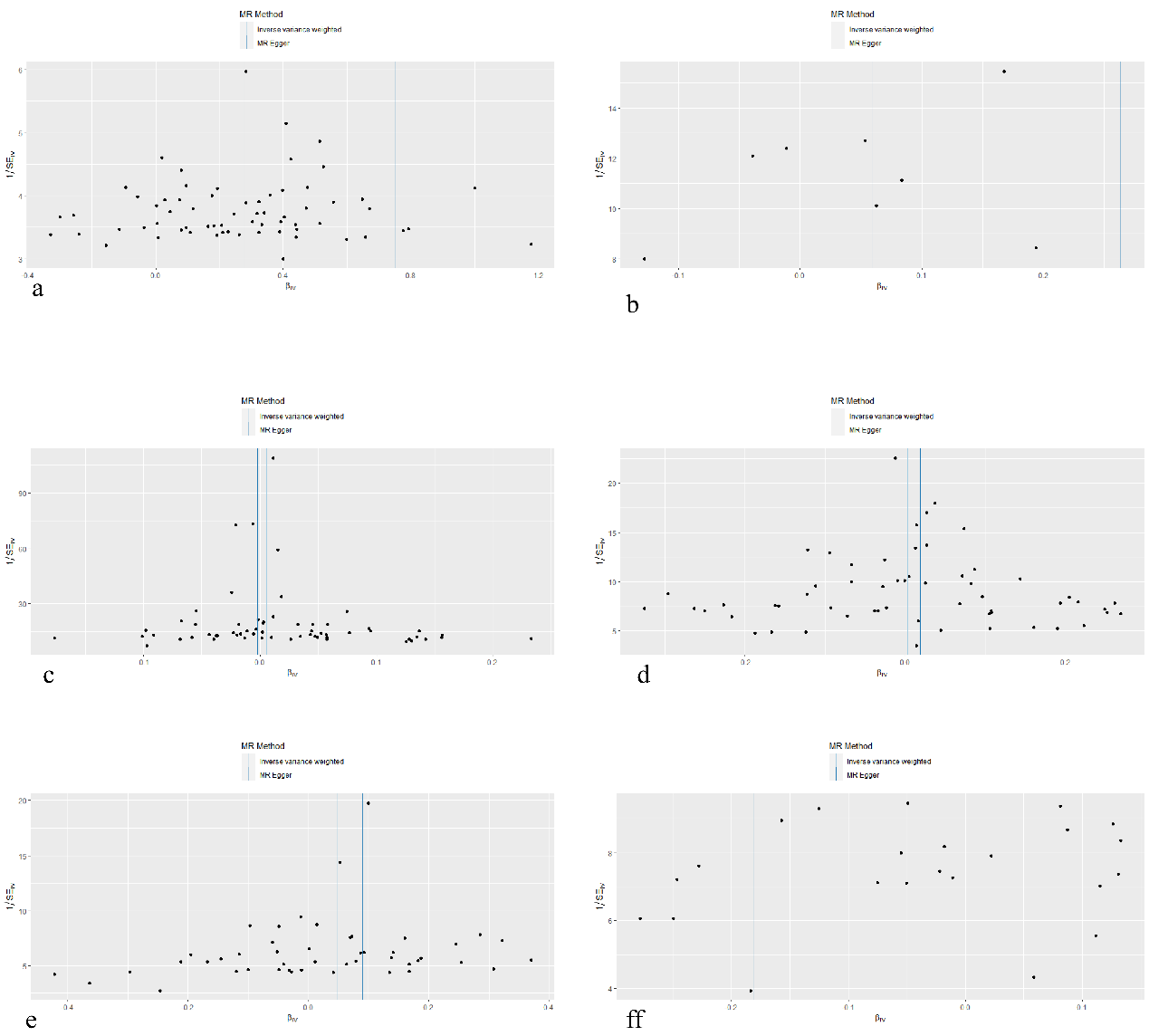


**Figure S11. Funnel plot of SNPs associated with gastrointestinal diseases and their risk of HF in the HERMES consortium. (a)** Gastroesophageal reflux disease. **(b)** Chronic gastritis. **(c)** Celiac disease. **(d)** Cholelithiasis with cholecystitis. **(e)** Non-alcoholic fatty liver disease. **(f)** Acute appendicitis.


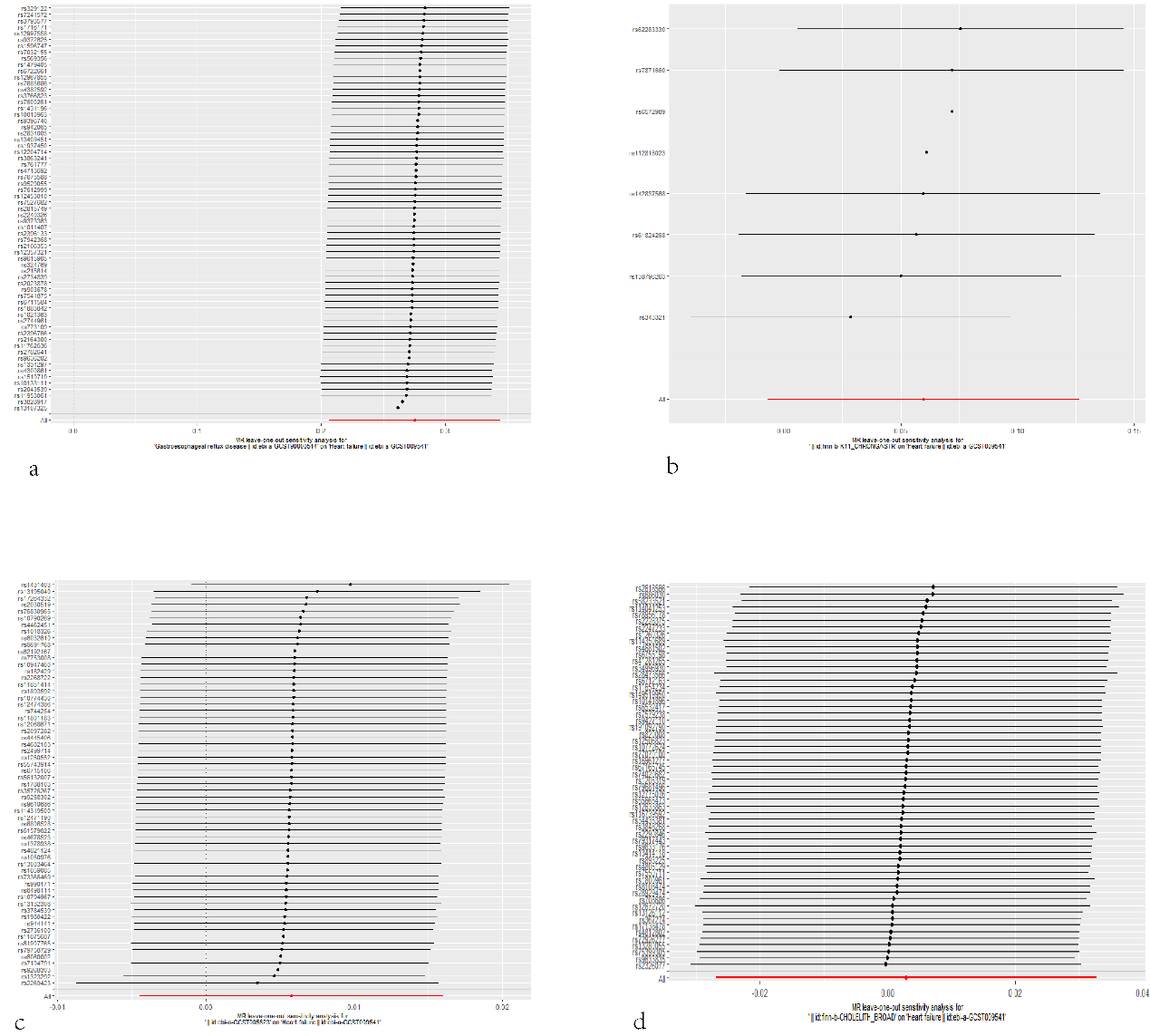


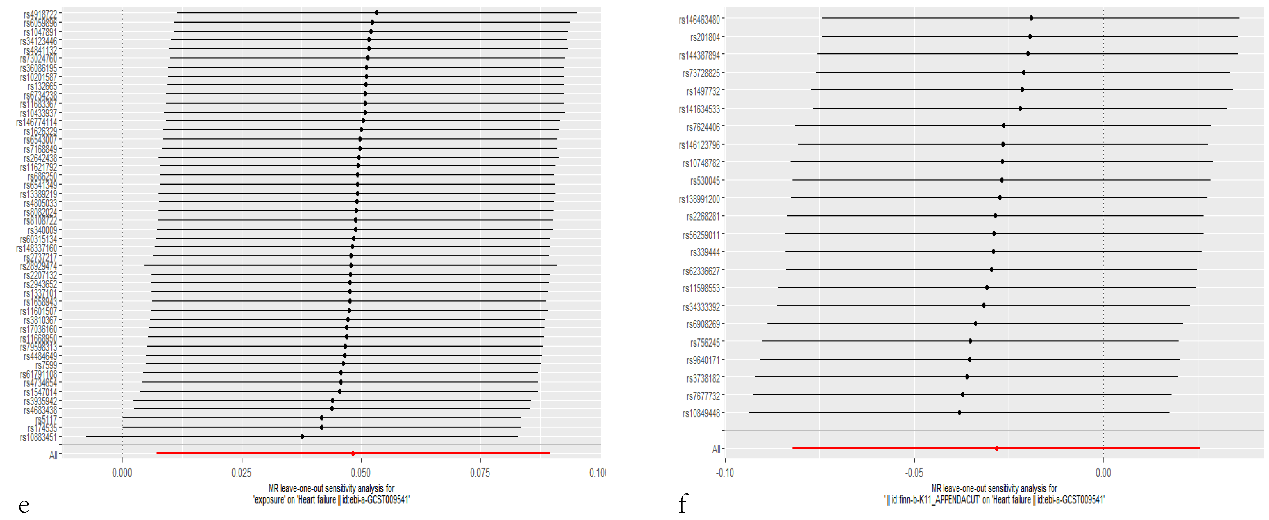


**Figure S12. Leave-one-out analysis of SNPs associated with gastrointestinal diseases and their risk of HF in the HERMES consortium.** **(a)** Gastroesophageal reflux disease. **(b)** Chronic gastritis. **(c)** Celiac disease. **(d)** Cholelithiasis with cholecystitis. **(e)** Non-alcoholic fatty liver disease. **(f)** Acute appendicitis.


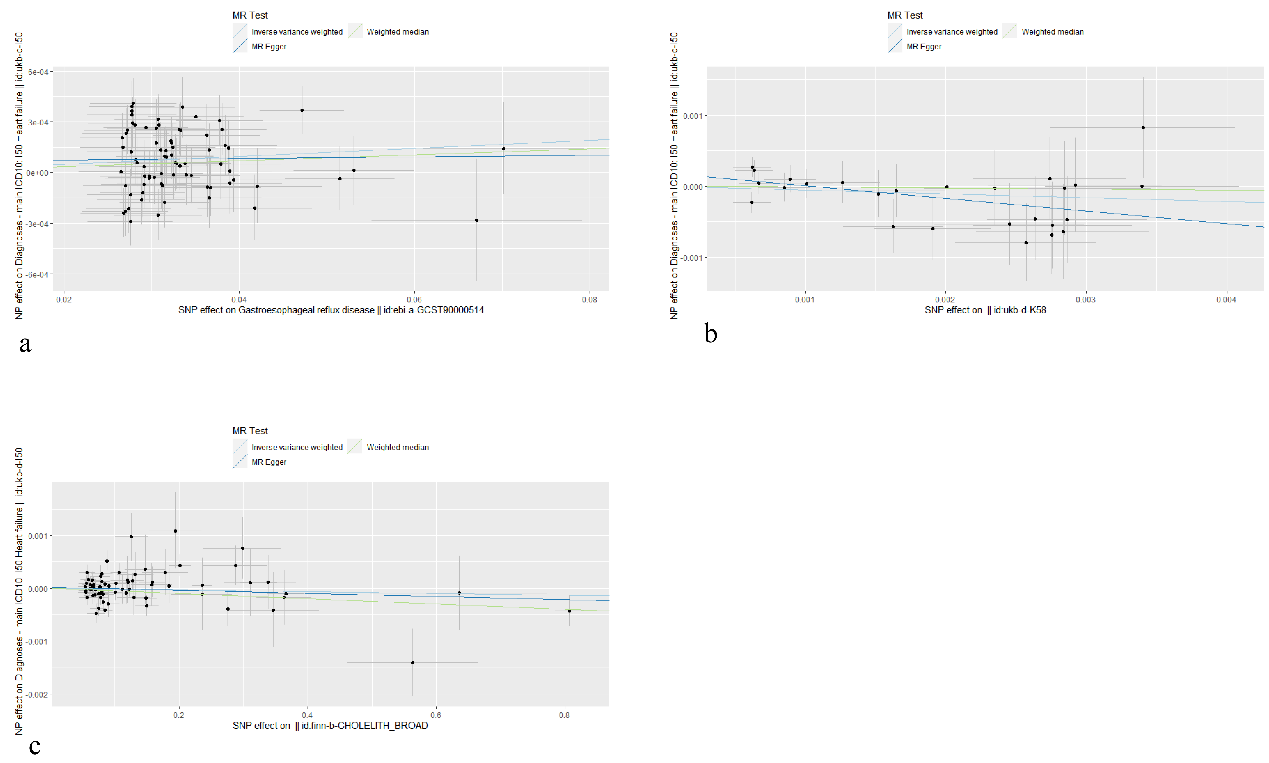


**Figure S13. Scatter plot of SNPs associated with gastrointestinal diseases and their risk of HF in UK Biobank.** **(a)** Gastroesophageal reflux disease. **(b)** Irritable bowel syndrome. **(c)** Cholelithiasis with cholecystitis.


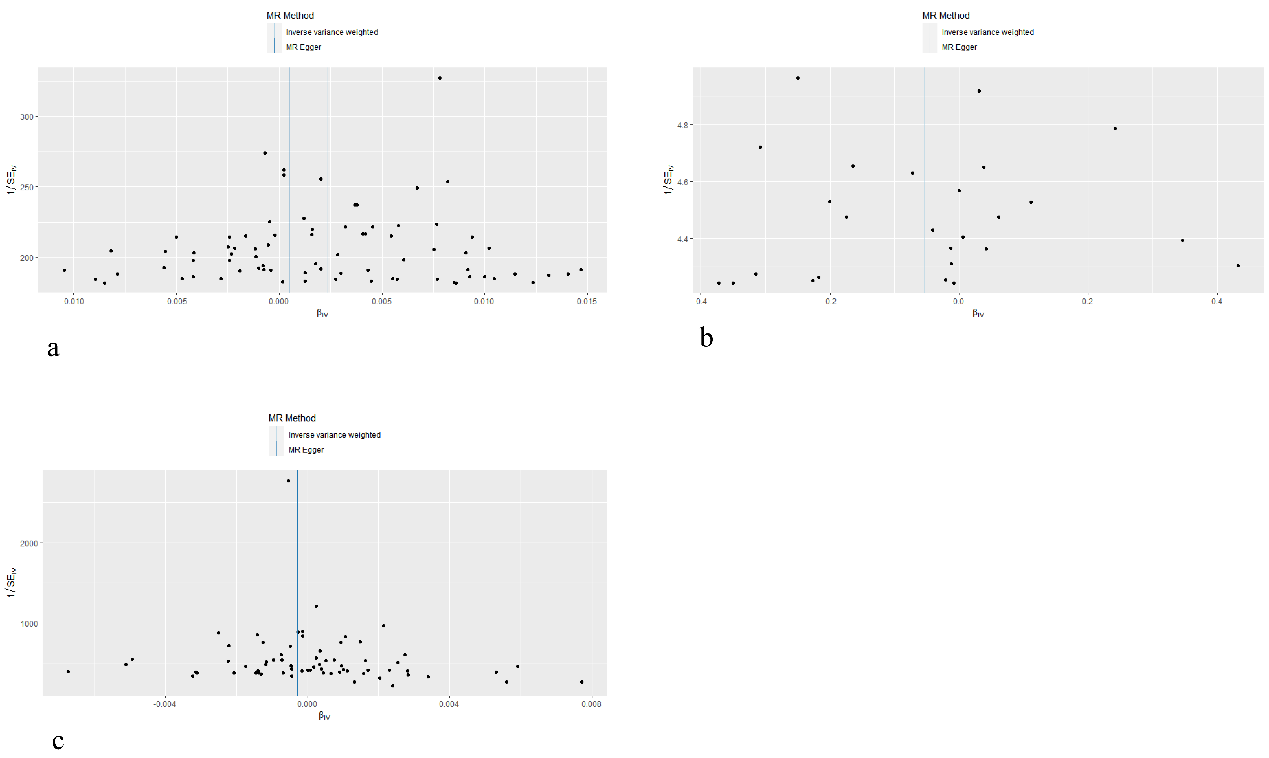


**Figure S14. Funnel plot of SNPs associated with gastrointestinal diseases and their risk of HF in UK Biobank.** **(a)** Gastroesophageal reflux disease. **(b)** Irritable bowel syndrome. **(c)** Cholelithiasis with cholecystitis.


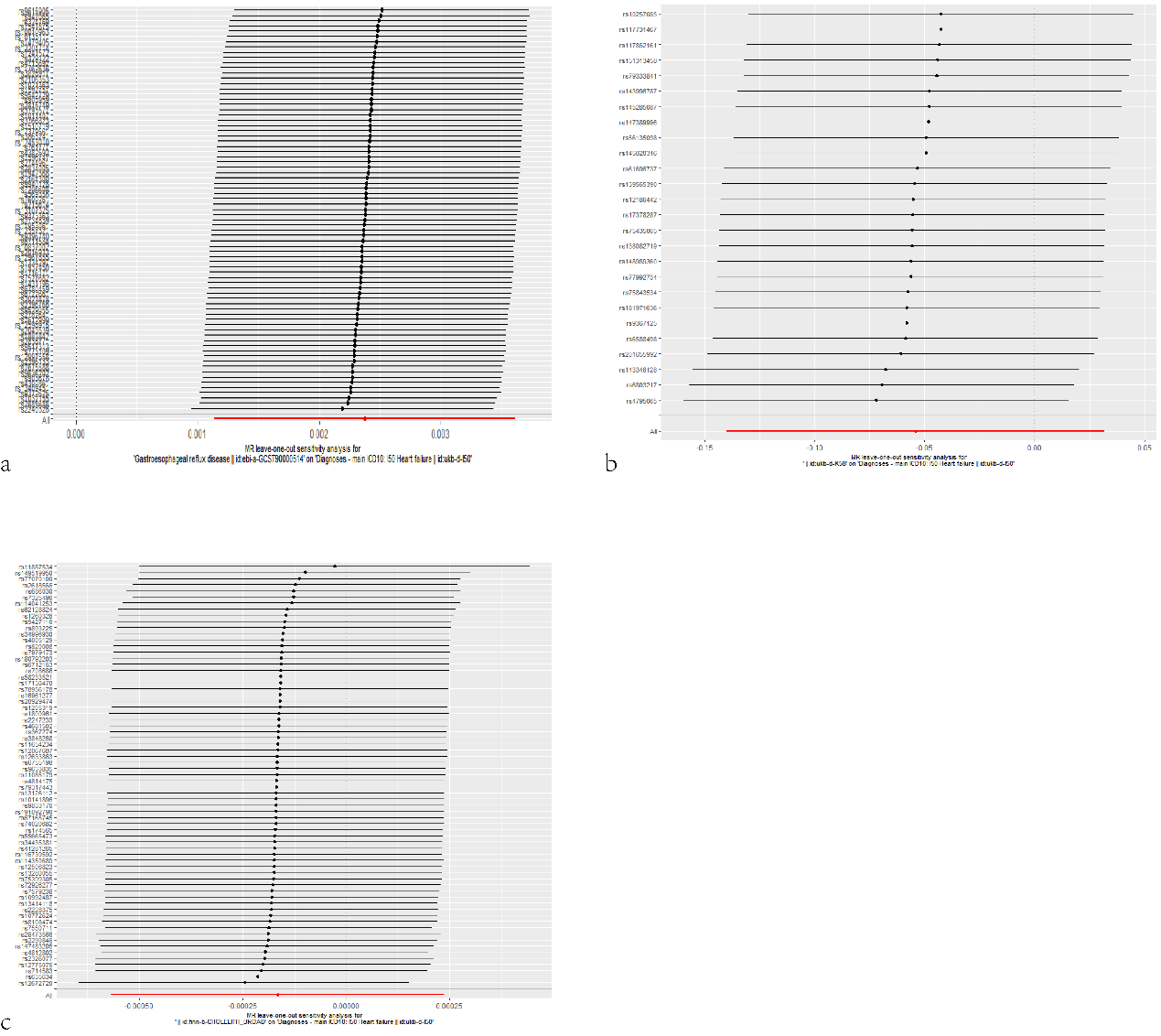


**Figure S15. Leave-one-out analysis of SNPs associated with gastrointestinal diseases and their risk of HF in FinnGen. (a)** Gastroesophageal reflux disease. **(b)** Irritable bowel syndrome. **(c)** Cholelithiasis with cholecystitis.


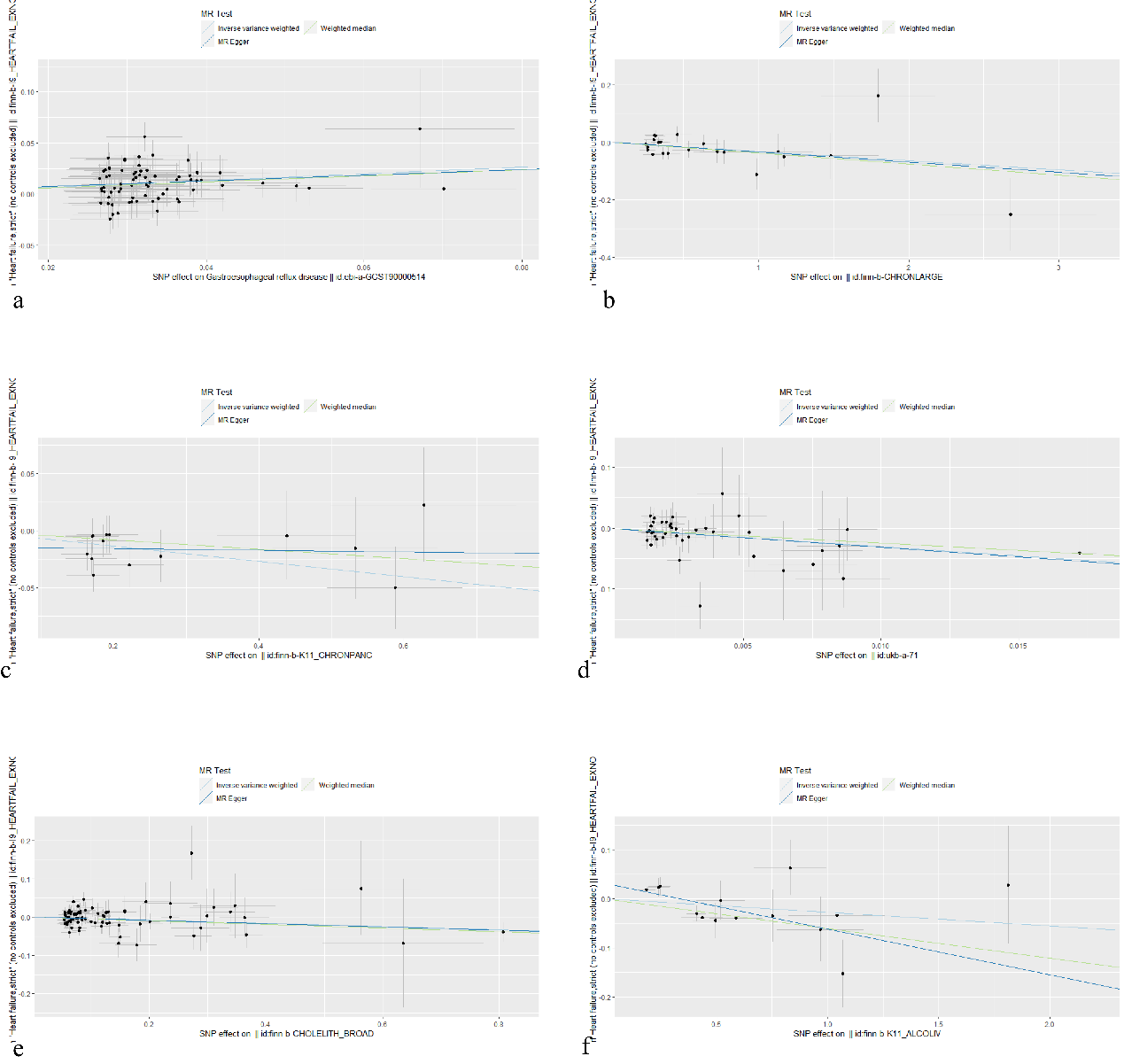


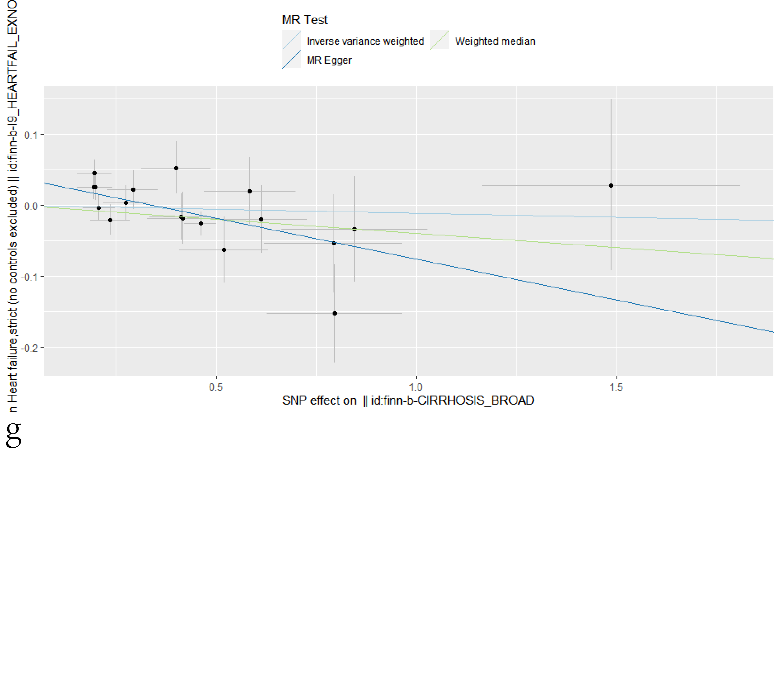


**Figure S16. Scatter plot of SNPs associated with gastrointestinal diseases and their risk of HF in FinnGen. (a)** Gastroesophageal reflux disease. **(b)** Crohn's disease. **(c)** Chronic pancreatitis. **(d)** Cholelithiasis. **(e)** Cholelithiasis with cholecystitis. **(f)** Alcoholic liver disease. **(g)** Liver cirrhosis.


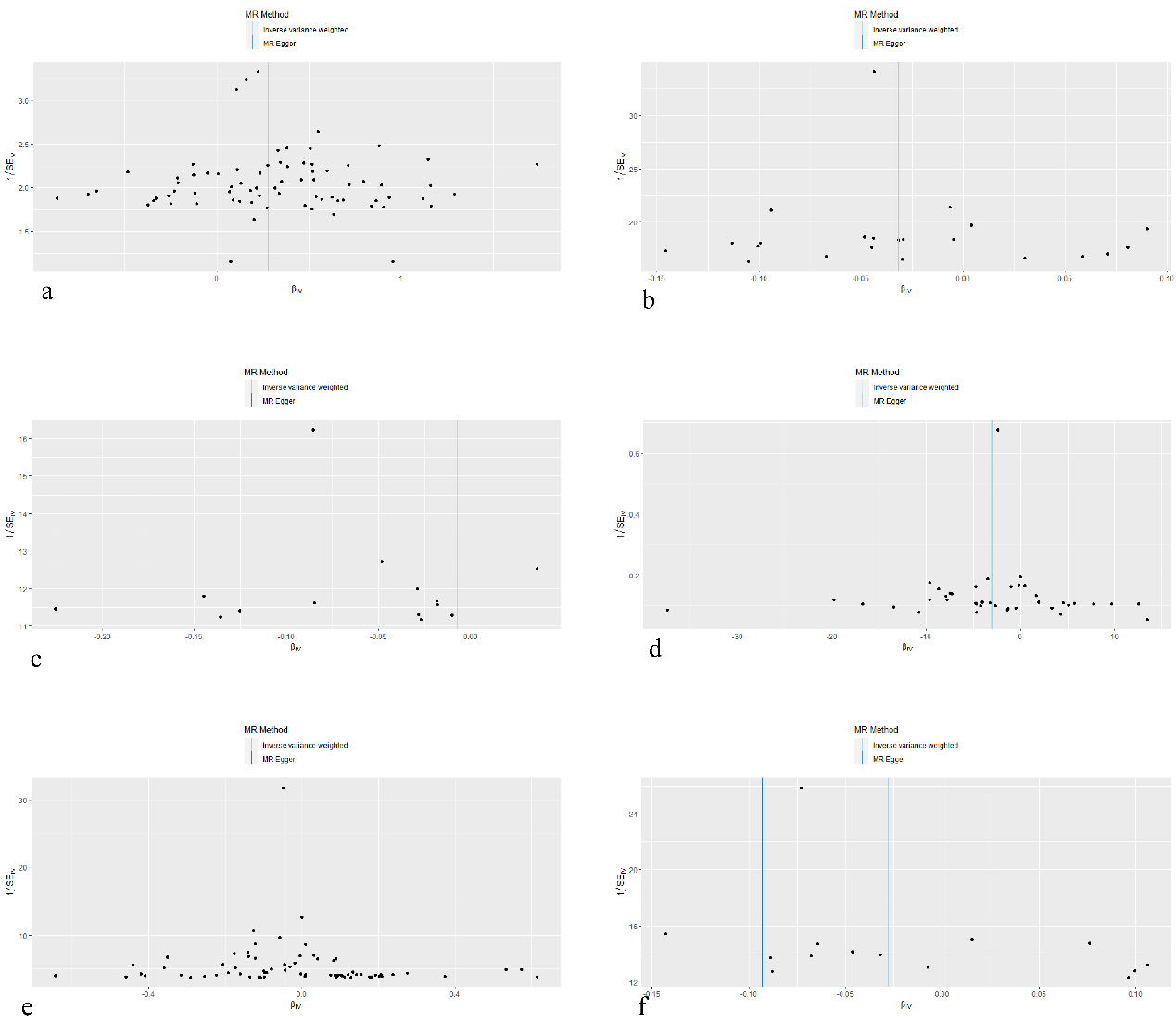


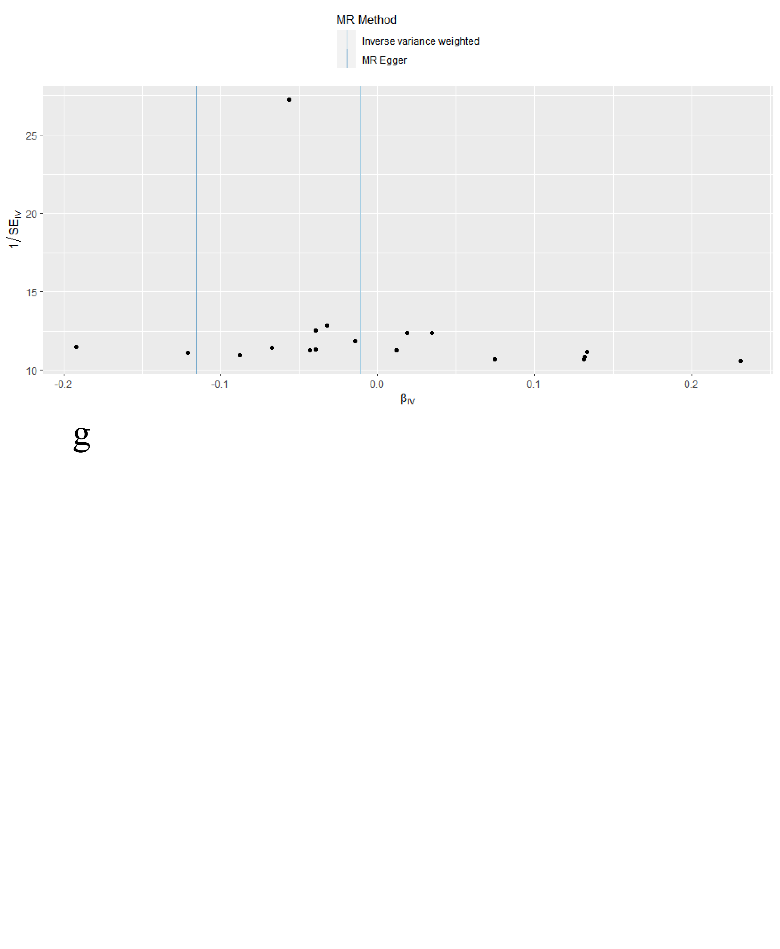


**Figure S17. Funnel plot of SNPs associated with gastrointestinal diseases and their risk of HF in FinnGen.** **(a)** Gastroesophageal reflux disease. **(b)** Crohn's disease. **(c)** Chronic pancreatitis. **(d)** Cholelithiasis. **(e)** Cholelithiasis with cholecystitis. **(f)** Alcoholic liver disease. **(g)** Liver cirrhosis.


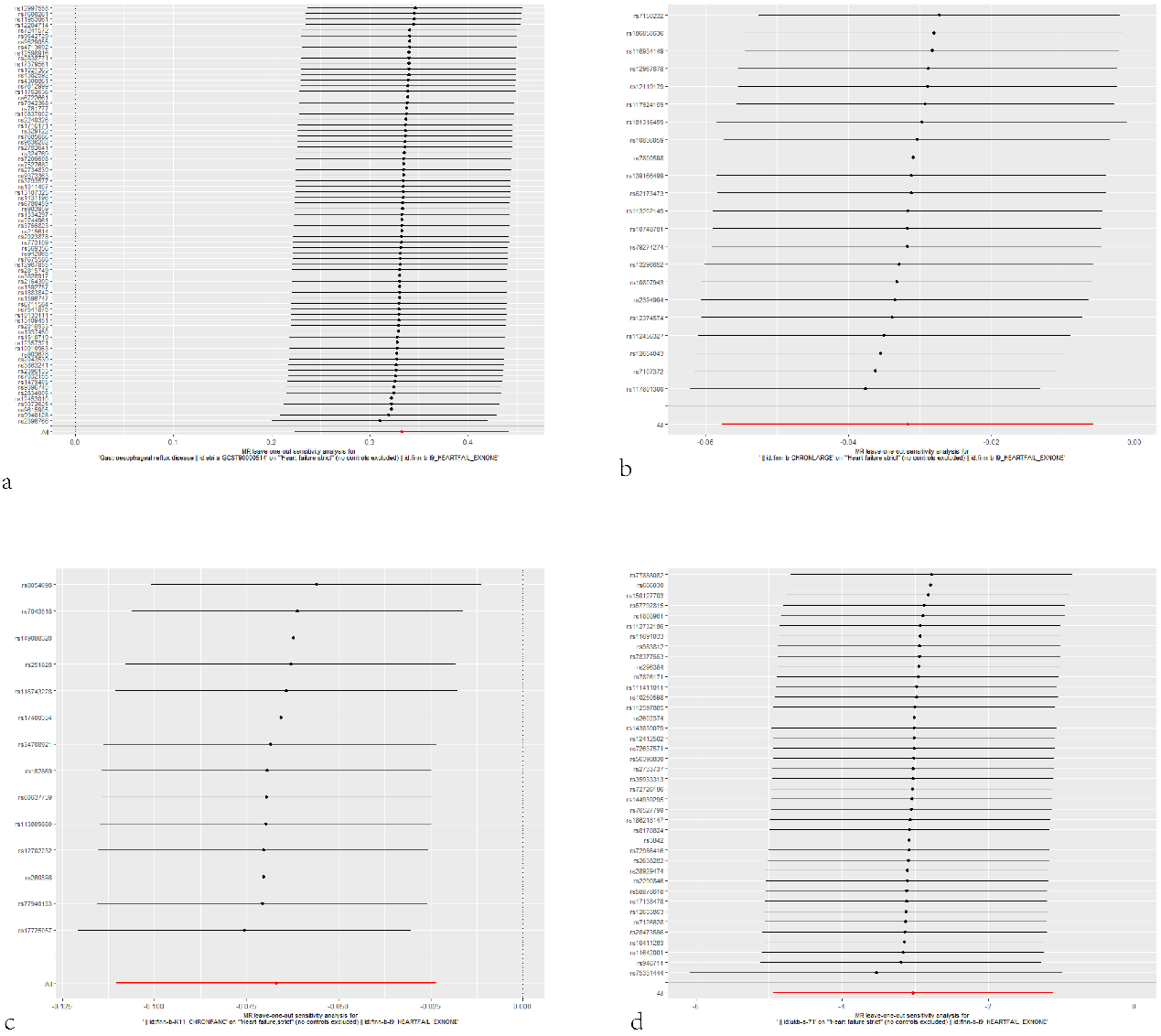


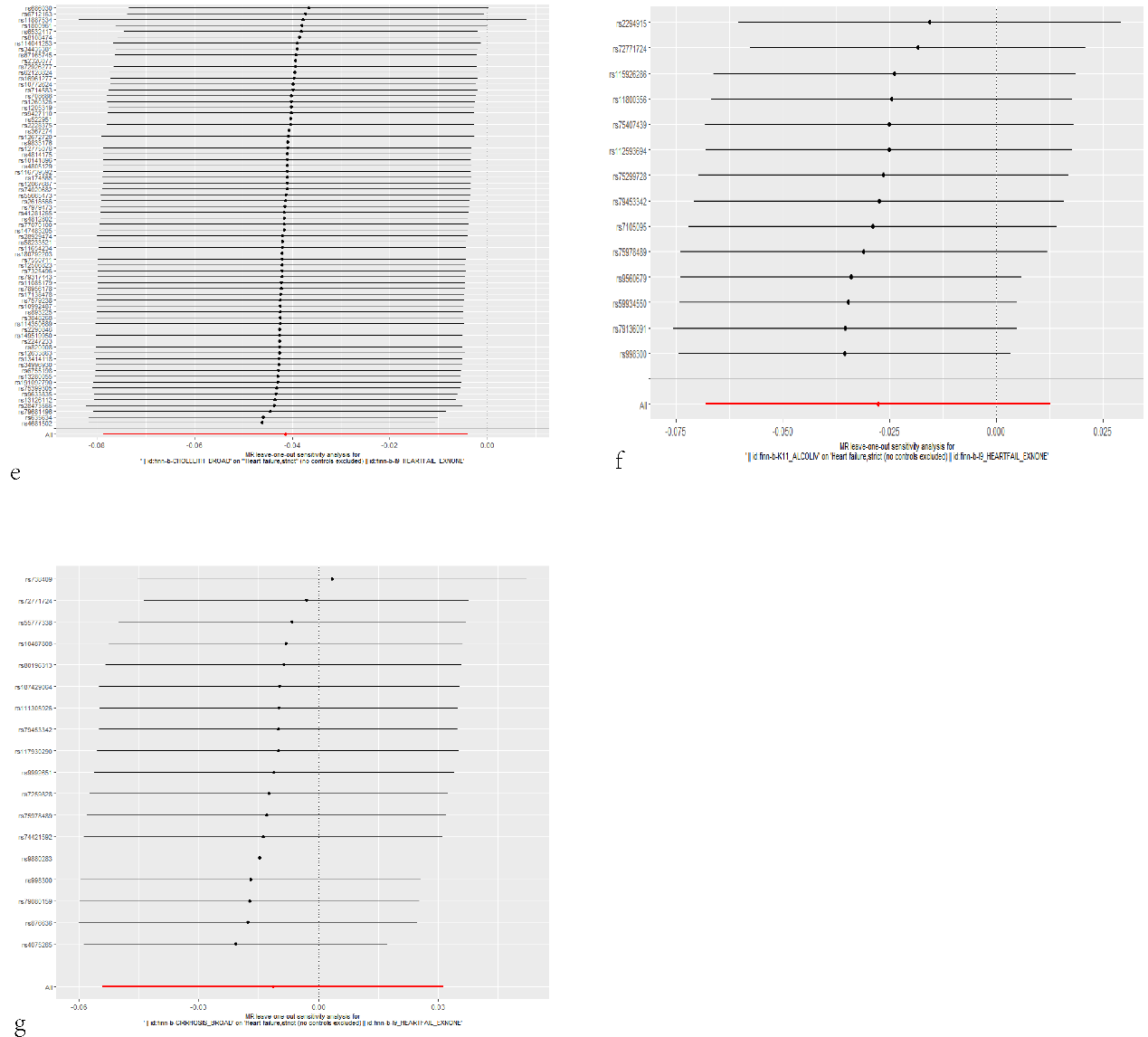


**Figure S18. Leave-one-out analysis of SNPs associated with gastrointestinal diseases and their risk of HF in FinnGen.** **(a)** Gastroesophageal reflux disease. **(b)** Crohn's disease. **(c)** Chronic pancreatitis. **(d)** Cholelithiasis. **(e)** Cholelithiasis with cholecystitis. **(f)** Alcoholic liver disease. **(g)** Liver cirrhosis.
