# Supplementary material for: Causal association between gastrointestinal diseases and coronary artery disease: a bidirectional Mendelian randomization study
Source: Front Endocrinol (Lausanne). 2024 Oct 15;15:1458196. doi: 10.3389/fendo.2024.1458196 (PMC11518705; doi:10.3389/fendo.2024.1458196)
Supplement: Supplementary file 2 [file DataSheet2.docx]

**STROBE-MR checklist of recommended items to address in reports of Mendelian randomization studies**^1^ ^2^

| **Item No.** | **Section** | **Checklist item** | **Page No.** | **Relevant text from manuscript** |
| --- | --- | --- | --- | --- |
| 1 | **TITLE and ABSTRACT** | Indicate Mendelian randomization (MR) as the study’s design in the title and/or the abstract if that is a main purpose of the study | Title | Causal association between gastrointestinal diseases and coronary artery disease: a bidirectional Mendelian randomization study  Abstract  Background: Coronary artery disease (CAD) has been a dominating reason of mortality globally due to its complexity of etiology. A variety of gastrointestinal disorders (GDs) have been accounted to be related to CAD. Thus, this study aims to determine their causal relationship by two-sample Mendelian randomization (MR) analysis.  Methods: Single-nucleotide polymorphisms (SNPs) relevant to 22 GDs were employed as instrumental variables from the genome-wide association summary (GWAS) datasets. Genetic associations with CAD and HF were acquired from UK Biobank, FinnGen, and other GWAS studies. We conducted a univariable MR (UVMR) analysis followed by a meta-analysis. A multivariable MR (MVMR) analysis was then performed with smoking and body mass index (BMI) as justifications. Also, a bi-directional MR analysis was leveraged to verify the reverse causal correlations.  Results: Generally, UVMR analyses separately observed the causal effects of GDs on CAD and HF. Genetic liability to gastroesophageal reflux disease displayed a positive association with both CAD (OR=1.19; 95%CI: 1.01-1.41) and HF (OR=1.22; 95%CI: 1.00-1.49) risk; genetic liability to celiac disease separately attributed to CAD (OR=1.02; 95%CI: 1.01-1.03) and HF (OR=1.01; 95%CI: 1.00-1.02), which also maintained after MVMR analysis. Besides, we observed mutually causal associations between CAD and celiac disease.  Conclusion: Our work suggested that genetic susceptibility to some GDs might causally increase the risk of CAD and HF, emphasizing the importance of preventing CAD in patients with GDs. |
|  | **INTRODUCTION** |  |  |  |
| 2 | **Background** | Explain the scientific background and rationale for the reported study. What is the exposure? Is a potential causal relationship between exposure and outcome plausible? Justify why MR is a helpful method to address the study question | Introduction | Coronary artery disease (CAD) was found to affect approximately 197 million patients and 9.1 million deaths, accounting for 16.1% of all deaths in 2019 (Collaborators, 2020). Considering the intricate pathogenesis of CAD, screening causal risk factors and preventing them is imperative, which is meaningful for attenuating the burden of related public health services (Malakar et al., 2019).  Currently, gastrointestinal diseases (GDs) are high-profile, and accumulating evidence suggests that they are responsible for multi-system disorders, such as Alzheimer's disease in the enteric nervous system (Ubhi and Masliah, 2013), depression of psychological aspect (Bisgaard et al., 2022), metabolic syndrome (Du et al., 2018), and cardiovascular diseases (Gesualdo et al., 2016). Population-based studies from epidemiology have observed that coronary atherosclerosis might be caused by nonalcoholic fatty liver disease and liver cirrhosis (Anstee et al., 2018, Wiese et al., 2014); gastroesophageal reflux disease (GERD) has been reported to cause angina and cardiac rhythm disturbances (Kato et al., 2009); ulcerative colitis to result in increased morbidity for CAD events (Kristensen et al., 2013). However, these observational causal connections are only sometimes consistent, particularly in the case of cardio-hepatic comorbidity. For example, a population-based study found that probably because the gluten-free recipe lowered blood pressure and cholesterol levels, celiac disease decreased the onset of myocardial infarction slightly (West et al., 2004).  The emerging MR analysis, supported by large-scale genome-wide association studies (GWAS) statistic data, has an advantage in identifying the etiogenic inference between risk agents and disease events by using genetic loci as instrumental variables (IVs) for exposure (Lawlor et al., 2008). Unlike conventional epidemiological methods, the MR approach exhibits merit in averting bias with confusion as alleles are assorted randomly when fertilizing instead of being confused by postnatal and self-developed factors (Davies et al., 2018). Also, MR analysis holds back an inverse relationship due to the lack of modification of germline genotype by disease (Pingault et al., 2018). Although published MR studies have reported the cause of several GDs, including GERD (Sun et al., 2022), celiac disease (Huang, 2022), and inflammatory bowel disease (Qiu et al., 2022), on the risk of CAD, these shreds of evidence were largely independent. |
| 3 | **Objectives** | State specific objectives clearly, including pre-specified causal hypotheses (if any). State that MR is a method that, under specific assumptions, intends to estimate causal effects | Introduction | In this article, we aimed to comprehensively inspect the associations of GDs with CAD by performing a two-sample MR study in both directions, followed by a meta-analysis. Additionally, according to updated data from the Framingham Heart Study, CAD is a major risk factor for HF (Lala and Desai, 2014). Thus, we examined the relationships between GDs and HF as supplementary evidence. Finally, we carried out a multivariable MR (MVMR) analysis to justify conventional predisposing agents. |
|  | **METHODS** |  |  |  |
| 4 | **Study design and data sources** | Present key elements of the study design early in the article. Consider including a table listing sources of data for all phases of the study. For each data source contributing to the analysis, describe the following: |  |  |
|  | a) | Setting: Describe the study design and the underlying population, if possible. Describe the setting, locations, and relevant dates, including periods of recruitment, exposure, follow-up, and data collection, when available. | Methods | Study design  This MR analysis was designed to examine the causal effect of common GDs on the risk of CAD and HF (Figure 1). We first performed a univariable MR (UVMR) analysis using 22 kinds of GDs and CAD as well as HF GWAS summary data of European populations, publicly available in the UK Biobank (UKB) study (Sudlow et al., 2015), FinnGen study, and other prominent international consortia. Then, we combined the findings from the above MR studies with a meta-analysis. We also conducted a bi-directional MR analysis to estimate the inverse causation. Finally, we verified the causal factors irrelevant to traditional risk elements by MVMR analysis, including smoking and body mass index (BMI). |
|  | b) | Participants: Give the eligibility criteria, and the sources and methods of selection of participants. Report the sample size, and whether any power or sample size calculations were carried out prior to the main analysis | Methods | We collected 22 GDs and assigned them to four broad location groups, including six upper GDs, six lower GDs, nine hepato-biliary and pancreas diseases, and others. Detailed data sources information for GDs was collected in Additional file 1: Table 1.  The GWAS statistical data for CAD was obtained from the Coronary Artery Disease Genome-wide Replication and Meta-analysis plus The Coronary Artery Disease Genetics (CARDIoGRAMplusC4D) consortium, comprising 6 0801 cases and 12 3504 controls from 48 individual studies (Nikpay et al., 2015). The summarized statistical data for HF in European ancestry was acquired from the Heart Failure Molecular Epidemiology for Therapeutic Targets (HERMES) consortium, including 47 309 samples and 930 014 controls(Shah et al., 2020). We further collected genetic information for CAD and HF from the UKB and FinnGen studies to conduct replication analyses, respectively. |
|  | c) | Describe measurement, quality control and selection of genetic variants | Methods | Therefore, single-nucleotide polymorphisms (SNPs) were selected through a series of steps as valid IVs. First, due to the limitation of numbers of SNPs satisfying the significant genome-wide, we appropriately widened the associated boundary with p < 1 × 10–6 to obtain top associated SNPs, except for GERD using P < 5×10-8. Second, those SNPs with linkage disequilibrium (LD) (r 2 > 0.001 and clump distance < 10 MB) were eliminated to acquire top independent SNPs. Meanwhile, we calculated the F statistics of individual SNP to prevent weak IVs, which was greater than the threshold of 10 (Lawlor et al., 2008). Then, we extracted the acquired SNPs associated with exposure from the outcome datasets. For SNPs unavailable in CAD outcomes, we searched proxies in high LD (r2 > 0.8) in the European reference panel of the 1000 Genomes Project (Machiela and Chanock, 2015), and we discarded those still unavailable through proxy. Finally, we coordinated the SNP alleles, which were associated not only with exposure but also with outcome. The procedures for selecting IVs in reverse MR analysis were the same. Applicable SNPs used in this study are shown in Additional file 1: Tables 2-4. |
|  | d) | For each exposure, outcome, and other relevant variables, describe methods of assessment and diagnostic criteria for diseases | Methods | The UKB research is a large-scale, promising cohort study with more than 500,000 subjects recruited from individual centers across the UK from 2006 to 2010. Summary level data for genetically related CAD in this study were available from UKB, involving 17655 cases defined by the codes I21 and I22 from the International Classification of Diseases version 10 (ICD-10). We further collected the European GWAS statistics for HF in UKB with the code I50 in ICD-10, comprising 1088 cases and 360 106 controls launched by the Neale Lab (Sudlow et al., 2015).  The FinnGen consortium aims to study genetic information with 30,952 samples and 187,840 controls from Finnish ancestry. We used the fifth release of the GWAS data on CAD and HF, diagnosed by the ICD-10 (Krawczyk and Święcicki, 2020). Detailed information on data sources and SNPs used as IVs are displayed in Additional file 1: Tables 1-4. |
|  | e) | Provide details of ethics committee approval and participant informed consent, if relevant | Methods | We would like to appreciate the CARDIoGRAMplusC4D consortium and the HERMES consortium for sharing the GWAS summary statistics for CAD and HF, and other large consortia including the UK Biobank study, FinnGen study, and consortia related to gastrointestinal diseases, and researchers for making the GWAS statistical data publicly available. |
| 5 | **Assumptions** | Explicitly state the three core IV assumptions for the main analysis (relevance, independence and exclusion restriction) as well assumptions for any additional or sensitivity analysis | Methods | The criterion for selecting eligible IVs for GDs needs to be met the following three hypotheses: (1) related to the exposure, namely relevance hypothesis; (2) uncorrelated with any confounding factors, namely independence hypothesis; (3) unconcerned with the outcome under exposure conditions, namely exclusion restriction hypothesis (Lawlor et al., 2008). |
| 6 | **Statistical methods: main analysis** | Describe statistical methods and statistics used |  |  |
|  | a) | Describe how quantitative variables were handled in the analyses (i.e., scale, units, model) | Methods | In this MR study, we applied UVMR analysis to evaluate the causal role of genetic liability to GDs on the risk of CAD and HF. We employed the inverse variance weighted (IVW) as the primary analysis, which produces the most accurate estimates but is liable to be biased by pleiotropy due to the assumption that all genetic instruments are valid (Bowden et al., 2017). We also used weighted median and MR-Egger methods to conduct sensitivity analyses, purposing to inspect the stability of the causality (Bowden et al., 2016, Bowden et al., 2015). The weighted median assumes that at least 50% of the SNPs are valid and provide the weight of estimates, while MR-Egger regression assumes that all SNPs are invalid. MR Pleiotropy Residual Sum and Outlier (MR-PRESSO) method can detect underlying horizontal pleiotropy (Verbanck et al., 2018) and correct for potential outliers contributing to heterogeneity across SNPs’ estimates (Verbanck et al.). We replicated primary and sensitivity analyses after discarding the outliers if significant pleiotropy was detected.  Finally, we conducted a meta-analysis of IVW estimates to identify the combined causality of GDs on CAD and HF from diverse sources. When there was pleiotropy, we selected the later MR results for the meta-analysis. Based on the I2 statistic, we used a fixed effect for I2 < 50%; otherwise, a random effect for I2 > 50% (Borenstein et al., 2010). The effects of meta-analysis were considered as the final causal references. Meanwhile, we were also concerned about other MR results that were only significant in a particular database. Furthermore, we conducted a reverse MR analysis if the causality was detected in the forward analysis, and the final results were also evaluated with a meta-analysis. |
|  | b) | Describe how genetic variants were handled in the analyses and, if applicable, how their weights were selected | Methods | We also performed the Cochran Q test and generated a funnel plot to appraise the heterogeneity and a leave-one-out (LOO) analysis to determine whether a specific SNP drove the pooled MR estimates (Verbanck et al., 2018). Besides, we adopted the Steiger directional test to determine the positive bi-directional causalities in forward MR analysis (Hemani et al., 2017). |
|  | c) | Describe the MR estimator (e.g. two-stage least squares, Wald ratio) and related statistics. Detail the included covariates and, in case of two-sample MR, whether the same covariate set was used for adjustment in the two samples | Methods | The MR results were presented as odds ratio (OR) and 95% confidence interval (CI). |
|  | d) | Explain how missing data were addressed | Methods | We replicated primary and sensitivity analyses after discarding the outliers if significant pleiotropy was detected. |
|  | e) | If applicable, indicate how multiple testing was addressed | Methods | Moreover, we applied the Benjamini-Hochberg approach to adjust for multiple testing. |
| 7 | **Assessment of assumptions** | Describe any methods or prior knowledge used to assess the assumptions or justify their validity |  |  |
| 8 | **Sensitivity analyses and additional analyses** | Describe any sensitivity analyses or additional analyses performed (e.g. comparison of effect estimates from different approaches, independent replication, bias analytic techniques, validation of instruments, simulations) | Methods | Finally, we conducted a meta-analysis of IVW estimates to identify the combined causality of GDs on CAD and HF from diverse sources. When there was pleiotropy, we selected the later MR results for the meta-analysis. Based on the I2 statistic, we used a fixed effect for I2 < 50%; otherwise, a random effect for I2 > 50% (Borenstein et al., 2010). The effects of meta-analysis were considered as the final causal references. Meanwhile, we were also concerned about other MR results that were only significant in a particular database. Furthermore, we conducted a reverse MR analysis if the causality was detected in the forward analysis, and the final results were also evaluated with a meta-analysis.  We also performed the Cochran Q test and generated a funnel plot to appraise the heterogeneity and a leave-one-out (LOO) analysis to determine whether a specific SNP drove the pooled MR estimates (Verbanck et al., 2018). Besides, we adopted the Steiger directional test to determine the positive bi-directional causalities in forward MR analysis (Hemani et al., 2017). Moreover, we applied the Benjamini-Hochberg approach to adjust for multiple testing. |
| 9 | **Software and pre-registration** |  |  |  |
|  | a) | Name statistical software and package(s), including version and settings used | Methods | All analyses were completed using “TwoSampleMR” (Hemani et al., 2018) and “MRPRESSO” R packages in RStudio Version 4.2.3 (Verbanck et al., 2018). |
|  | b) | State whether the study protocol and details were pre-registered (as well as when and where) | - | Not applicable. |
|  | **RESULTS** |  |  |  |
| 10 | **Descriptive data** |  |  |  |
|  | a) | Report the numbers of individuals at each stage of included studies and reasons for exclusion. Consider use of a flow diagram | Results | 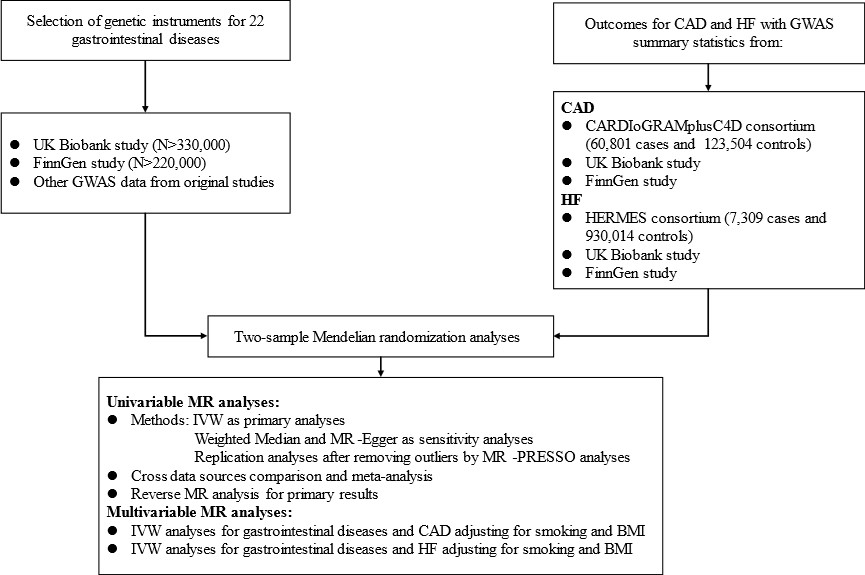 |
|  | b) | Report summary statistics for phenotypic exposure(s), outcome(s), and other relevant variables (e.g. means, SDs, proportions) | Results | The detailed informations for phenotypic exposures and outcomes are displayed in uploaded additional file: Tables 2-4. |
|  | c) | If the data sources include meta-analyses of previous studies, provide the assessments of heterogeneity across these studies | - | Not applicable. |
|  | d) | For two-sample MR:  i.  Provide justification of the similarity of the genetic variant-exposure associations between the exposure and outcome samples  ii.  Provide information on the number of individuals who overlap between the exposure and outcome studies | Results | Exposures and samples were drawn from separate large databases, so sample overlap was avoided. |
| 11 | **Main results** |  |  |  |
|  | a) | Report the associations between genetic variant and exposure, and between genetic variant and outcome, preferably on an interpretable scale | Results | First, due to the limitation of numbers of SNPs satisfying the significant genome-wide, we appropriately widened the associated boundary with p < 1 × 10–6 to obtain top associated SNPs, except for GERD using P < 5×10-8. Second, those SNPs with linkage disequilibrium (LD) (r 2 > 0.001 and clump distance < 10 MB) were eliminated to acquire top independent SNPs. Meanwhile, we calculated the F statistics of individual SNP to prevent weak IVs, which was greater than the threshold of 10 (Lawlor et al., 2008). Then, we extracted the acquired SNPs associated with exposure from the outcome datasets. For SNPs unavailable in CAD outcomes, we searched proxies in high LD (r2 > 0.8) in the European reference panel of the 1000 Genomes Project (Machiela and Chanock, 2015), and we discarded those still unavailable through proxy. Finally, we coordinated the SNP alleles, which were associated not only with exposure but also with outcome. The procedures for selecting IVs in reverse MR analysis were the same. Applicable SNPs used in this study are shown in Additional file 1: Tables 2-4. |
|  | b) | Report MR estimates of the relationship between exposure and outcome, and the measures of uncertainty from the MR analysis, on an interpretable scale, such as odds ratio or relative risk per SD difference | Results | 3 Results  3.1 Gastrointestinal Diseases and CAD  Hereditary susceptibility to 3 GDs were increasingly associated with risk of CAD in meta-analysis (Table 1 and Figure 2). For 1-unit increment in log-transformed OR of GERD and celiac disease, the combined OR was separately 1.19 (95%CI: 1.01-1.41; pmeta=0.0001) and 1.02 (95% CI: 1.01-1.03; pmeta=0.0284) for CAD risk, together duodenal ulcer with the same directional association (OR=0.99; 95%CI: 0.97-1.02; pmeta=0.0307).  Additionally, there were other causal references of GDs with CAD in some databases, but the causality lost significance after the combination. Genetic liability to gastric ulcer with OR=1.06 (95%CI: 1.00-1.12; p=0.047), Crohn's disease with OR=0.979 (95%CI: 0.963-0.997; p=0.02) and NAFLD with OR=1.14 (95%CI: 1.08-1.20; p=1.08E-06) was positively associated with CAD only in CARDIoGRAMplusC4D; pancreatic cancer with OR=1.10 (95%CI: 1.03-1.17; p=4.65E-03) and cholelithiasis with OR=0.05 (95%CI: 0.01-0.37; p=3.20E-03) only in FinnGen. All these associations, only the risk of GERD on CAD, remained significant after multiple tests (Additional file 1: Table 5).  3.2 Gastrointestinal Diseases and HF  Genetic susceptibility to 3 GDs was correlated to the risk of HF (Table 1 and Figure 2). With genetic prediction, per-unit increment in log-transformed OR of GERD was highly associated with HF (OR=1.22; 95% CI: 1.00-1.49; pmeta=0.0001) and celiac disease (OR=1.01; 95% CI: 1.00-1.02; pmeta=0.12), but the significant effect for celiac disease did not maintain in multiple testing. Meanwhile, there were associations for Crohn's disease (OR=0.97; 95% CI: 0.94-0.99; p=0.02), chronic pancreatitis (OR=0.94; 95%CI: 0.90-0.98; p=2.50E-03), cholelithiasis (OR=0.05; 95%CI: 0.01-0.33; p=1.93E-03), cholelithiasis with cholecystitis (OR=0.96; 95%CI: 0.92-1.00; p=0.03) with HF in FinnGen, and NAFLD (OR=1.05; 95%CI: 1.00-1.09; p=0.02) in HERMES. |
|  | c) | If relevant, consider translating estimates of relative risk into absolute risk for a meaningful time period | - | Not applicable. |
|  | d) | Consider plots to visualize results (e.g. forest plot, scatterplot of associations between genetic variants and outcome versus between genetic variants and exposure) | - | 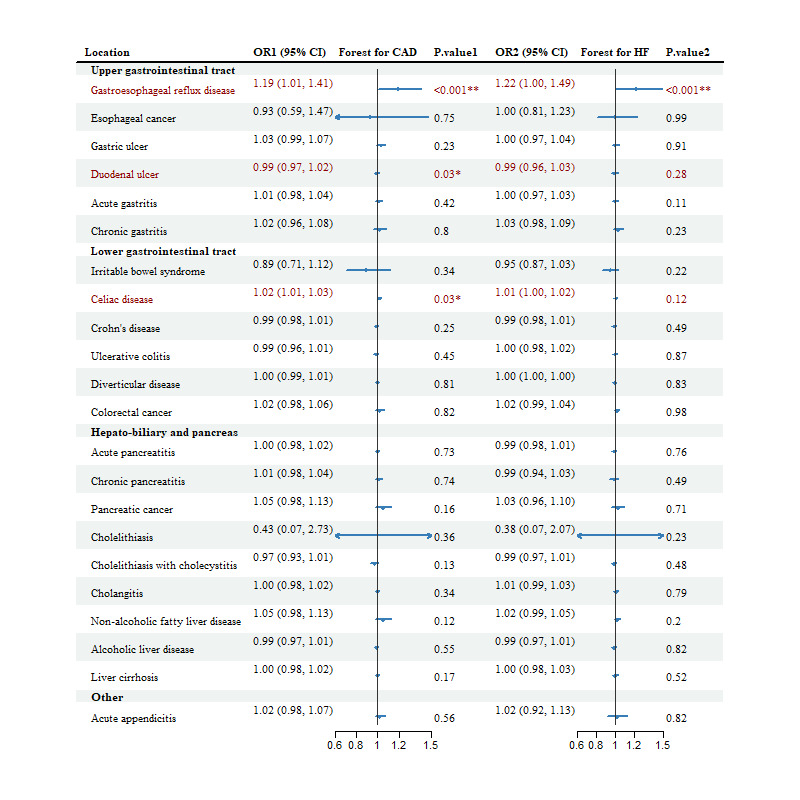  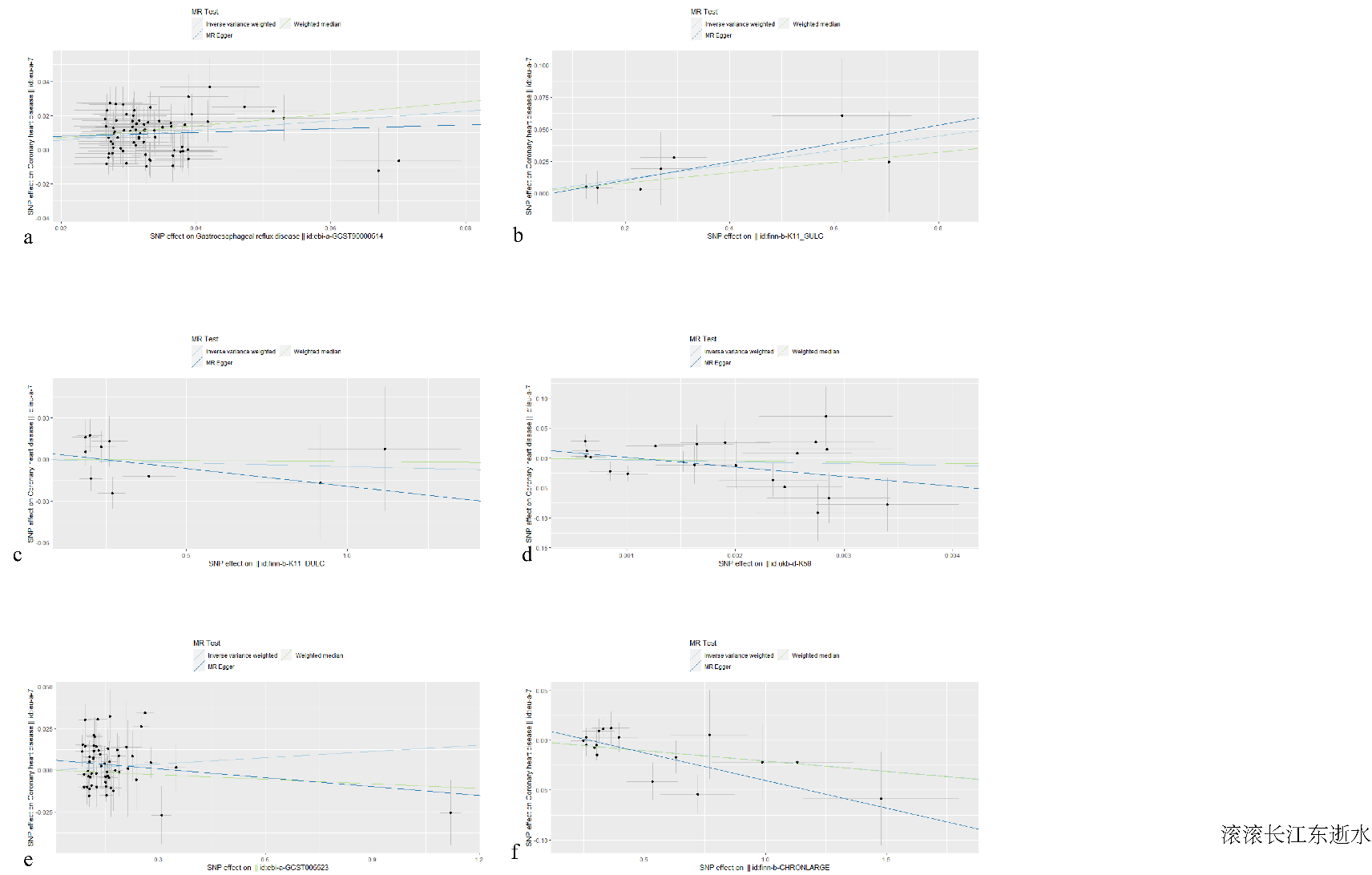 |
| 12 | **Assessment of assumptions** |  |  |  |
|  | a) | Report the assessment of the validity of the assumptions | Results | In short, we ensured the reliability of UVMR analyses in the following areas. First, the selected IVs were valid as the F statistics were above 10, except for esophageal cancer, irritable bowel syndrome, and cholelithiasis, suggesting bias from weak instrumentals (Additional file 1: Tables 3-4). |
|  | b) | Report any additional statistics (e.g., assessments of heterogeneity across genetic variants, such as *I^2^*, Q statistic or E-value) | Results | Second, although there was heterogeneity in Cochran’s Q test and pleiotropy in the MR-Egger examination for some causalities (Additional file 1: Tables 6, 10), we did not inspect severe asymmetry in funnel plots and significant Egger intercepts in scatter plots after removing the outliers. Further, no single SNP disturbed the combined effect of GDs on CAD and HF in LOO analysis (Additional file 2: Figures 1-18). |
| 13 | **Sensitivity analyses and additional analyses** |  |  |  |
|  | a) | Report any sensitivity analyses to assess the robustness of the main results to violations of the assumptions | Results | The causal effects of GDs on CAD were in accordance with primary results in sensitivity analyses in the same direction (Additional file 1: Table 6). We detected heterogeneity in several gastrointestinal outcomes. We also observed horizontal pleiotropy in the MR-Egger test for cholelithiasis and NAFLD. As for MR-PRESSO analysis, we identified one to 16 outliers in all three data sources, mainly for celiac disease, cholelithiasis, and NAFLD. These associations survived but with smaller estimates and more rigorous CIs after removing outliers except for that for cholelithiasis with no substantial risk of CAD.  These causal associations remained directionally unitive in sensitivity analyses; however, we still detected heterogeneity and horizontal pleiotropy in MR-Egger for some outcomes, such as GERD in HERMES. Besides, we detected a few outliers in MR-PRESSO in most consequences; despite smaller effect sizes, the causal effects survived after removing these outliers (Additional file 1: Table 10). |
|  | b) | Report results from other sensitivity analyses or additional analyses | Results | 3.3 Multivariable MR analysis  We further adjusted for smoking and BMI using MVMR analysis to rule out underlying pleiotropies (Additional file 1: Tables 12-13). It turned out that genetic liability to GERD still displayed the strongest association with CAD (OR=1.15; 95%CI: 1.00-1.33; pmeta=0.0001) for smoking and (OR=1.14; 95%CI: 1.00-1.31; pmeta =0.0001) for BMI, together with HF (OR=1.18; 95%CI: 1.00-1.41; pmeta =0.0019) for smoking and (OR=1.16; 95%CI: 1.00-1.37; pmeta =0.0008) for BMI. The causal effect of celiac disease on HF barely reached significance after correcting with smoking (OR=1.01; 95%CI: 1.00-1.02; pmeta=0.2405) and BMI (OR=1.00; 95%CI: 1.00-1.01; pmeta =0.4030), but acute appendicitis lost the significance, implying that we should treat these causalities with caution.  Intricately, when added to MVMR analysis, BMI exhibited an attractive influence on the association of colorectal cancer and CAD, which made the causality statistically positive (OR=0.9968; 95%CI: 0.9940-0.9996; pmeta=0.0263; beta=-0.0032). Furthermore, smoking also contributed to a significantly increased causal effect of irritable bowel syndrome on HF (OR=0.73; 95%CI: 0.56-0.97; pmeta =0.0285; beta=-0.3085). |
|  | c) | Report any assessment of direction of causal relationship (e.g., bidirectional MR) | Results | Reversely, no causal associations of CAD on GDs were detected, including GERD (OR=1.02; 95%CI: 0.99-1.04; pmeta=0.1356) and duodenal ulcer (OR, 1.00; 95%CI, 1.00-1.00; pmeta=0.2125) (Additional file 1: Table 7). Interestingly, CAD appeared to affect the onset of celiac disease (OR=1.24; 95%CI: 1.02-1.50; pmeta=0.0279), implying a bidirectional causal reference, which was also identified in Steiger’s directional test (Additional file 1: Table 8). The above results remained stable in sensitivity analyses and survived after adjustment in MVMR analysis (Additional file 1: Table 9).  We further detected bi-directional MR analysis of HF on these GDs, and no evidence suggesting a causal effect for GERD (OR=1.07; 95%CI: 0.97-1.19; pmeta=0.1959) and celiac disease (OR=1.21; 95%CI: 0.84-1.75; pmeta=0.2662). Besides, we did not observe horizontal pleiotropy except for NAFLD (Additional file 1: Table 11). |
|  | d) | When relevant, report and compare with estimates from non-MR analyses | Results | We cited both observational and experimental findings as references in the discussion section. |
|  | e) | Consider additional plots to visualize results (e.g., leave-one-out analyses) | - | We used the leave-one-out analyses, scatter plots and funnel plots to show our results, and the whole results are in the uploaded Appendix 2, and the following are some examples.  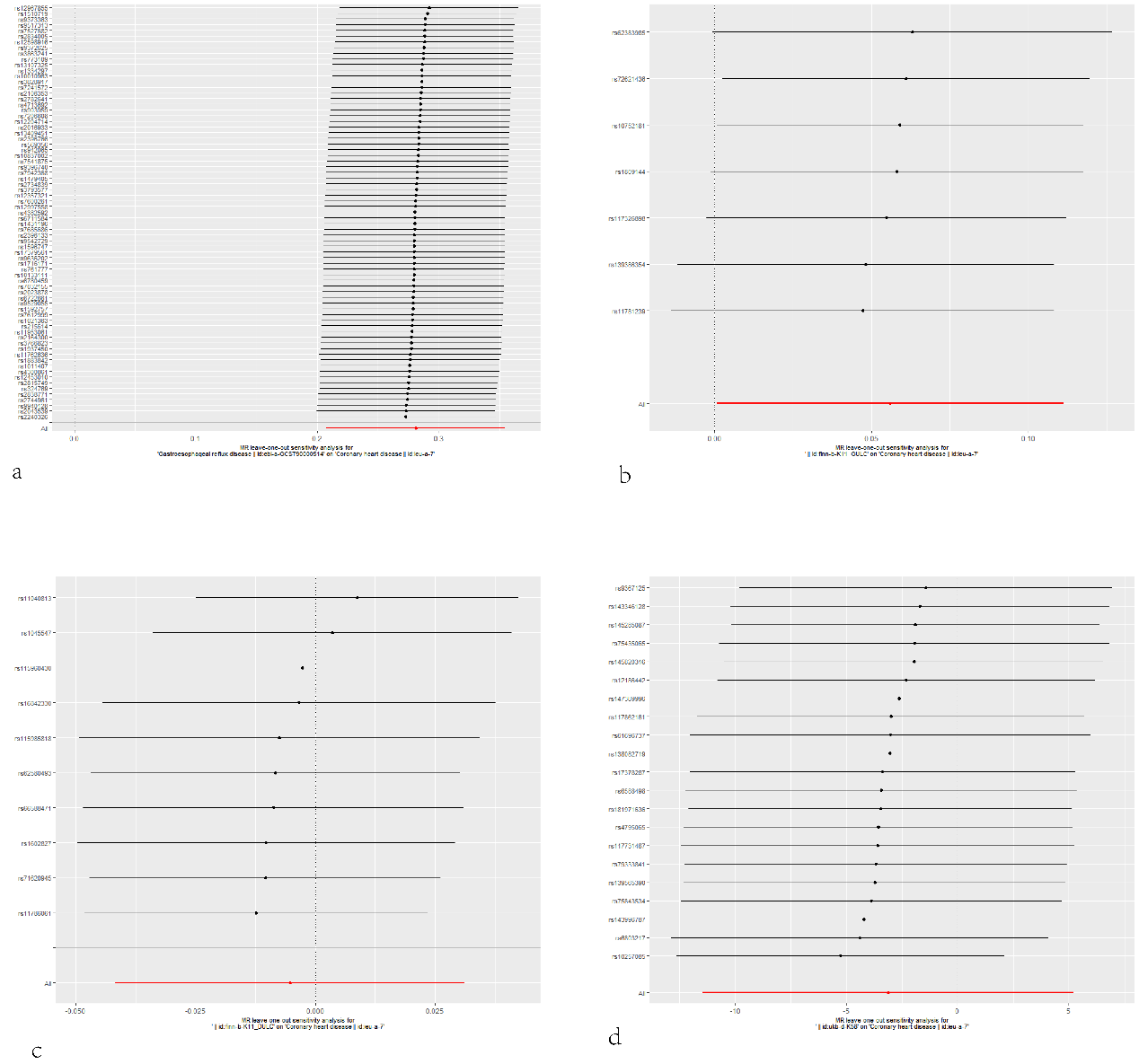 |
|  | **DISCUSSION** |  |  |  |
| 14 | **Key results** | Summarize key results with reference to study objectives | Discussion | Exploration and illustration of the causal risk factors of GDs on cardiovascular diseases, usually coexisting clinically, can inform the formulation of optimal prevention strategies early. This study estimated the causal association of 22 GDs with CAD and HF by the two-sample MR analysis and verified the results. By analyzing diverse large-scale GWAS datasets using an integrated MR analysis process, we confirmed several genetic liabilities to GDs that exerted a causal role on CAD and HF; meanwhile, we ruled out some of those that have traditionally been regarded as risk factors for CAD. |
| 15 | **Limitations** | Discuss limitations of the study, taking into account the validity of the IV assumptions, other sources of potential bias, and imprecision. Discuss both direction and magnitude of any potential bias and any efforts to address them | Discussion | Nonetheless, the work has several limitations: 1. Our study only employed data from European ancestry, which is likely slanted by the population structure bias. 2. Because the number of SNPs associated with exposure as IVs did not satisfy the significant genome-wide because of stringent screening conditions, we relaxed the threshold for P value, a common approach to deal with too few SNPs. 3. We adjusted the effect estimates for multiple testing via Benjamini-Hochgerg, which was a relatively relaxing way. However, we repeated the analysis in different datasets, largely avoiding false positives. 4. There were certain horizontal pleiotropies in MR-Egger, implying that GDs-related SNPs result in CAD through other traits. Still, we conducted MVMR analysis adjusting for smoking and BMI and replicated analyses after removing outliers detected in MR-PRESSO. 5. Owing to all exposures defined as binary phenotypes, the causations may be predetermined by the hypothesis of exclusion restriction (Burgess and Labrecque, 2018). However, GDs are not only diagnosed by continuous indicators but also by clinical symptoms and others, suggesting that we tried to uphold this assumption. In a word, we hope to resolve the deficiencies mentioned above in future studies with the emerging public availability of GWAS data in diverse regions worldwide and the demonstration of these causalities based on epidemiologic studies. |
| 16 | **Interpretation** |  |  |  |
|  | a) | Meaning: Give a cautious overall interpretation of results in the context of their limitations and in comparison with other studies | Discussion | As one of the most common GDs, GERD traditionally manifests as heartburn and acid regurgitation, yet it usually presents with atypical symptoms, such as epigastric pain and chest pain, that mimic those of CAD (Hunt et al., 2017); thus, it is difficult to distinguish between the two and contribute to misdiagnosis. To date, increasing epidemiological proofs observe a concurrence of GERD and CAD due to the utilization of proton pump inhibitors potentially (Dobrzycki et al., 2005). Therefore, early prevention of CAD in patients with GERD is necessary.  We also detected an increased risk between genetic liability to celiac disease and CAD, verified in epidemiology (Ludvigsson et al., 2011). Although the causality was not maintained in multiple testing and MVMR analysis, indicating that the causal role of celiac disease on CAD might be biased by smoking and BMI traits, our results were still more persuasive as using 56, 57, and 52 SNPs from three diverse GWAS data sources separately than those of Huang based on 15 SNPs from only one GWAS dataset (Huang, 2022). Notably, we observed a bidirectional relationship between celiac disease and CAD, which might attributed to the shared pathogenesis, such as anabatic systemic inflammation, triggering celiac disease in individuals with CAD (Danese et al., 2007). Moreover, the inverse causality was reserved after adjusting for smoking and BMI, and the effect sizes were even more significant than those in forward MR analysis. Overall, it is of importance clinically to promote the identification and therapy of celiac disease in CAD patients.  Furthermore, we observed a slightly increased risk of duodenal ulcer on CAD, but the causality may be false positive biased by heterogeneity from different GWAS data sources. Inversely, the finding from a prospective study was that patients with CAD were highly associated with duodenal ulcers due to the use of aspirin (Nema et al., 2009). Given this controversy in epidemiology and the slight significance of this study, the relationship between duodenal ulcer and CAD still needs to be explored.  We also found that genetic liability to celiac disease is causally associated with HF with a very slight significance, and the point estimate was mildly above 1, suggesting that the causality might be expected just by chance. Unexpectedly, celiac disease was seemingly relevant with a diminished encumbrance of traditional risk factors for cardiovascular diseases, such as higher BMI and incidence of type 2 diabetes, and a smaller likelihood of smoking (Conroy et al., 2023), which explained why the effect sizes did not differ much before and after adjustment. |
|  | b) | Mechanism: Discuss underlying biological mechanisms that could drive a potential causal relationship between the investigated exposure and the outcome, and whether the gene-environment equivalence assumption is reasonable. Use causal language carefully, clarifying that IV estimates may provide causal effects only under certain assumptions | Discussion | This work has several strengths based on the intrinsic study mechanism. First is the study design derived from the MR method, averting bias with confusion as alleles are assorted randomly when fertilizing instead of being confused by postnatal and self-developed factors, and we integrally analyzed the causal effects across 22 GDs on CAD and HF. Second, the dependability derives from applying the summary level data of the enormous GWAS datasets and subsequently following a meta-analysis and a bi-directional MR analysis to elucidate the anastrophic causation. We also conducted an MVMR analysis to mitigate horizontal pleiotropy caused by other underlying factors. |
|  | c) | Clinical relevance: Discuss whether the results have clinical or public policy relevance, and to what extent they inform effect sizes of possible interventions | Discussion | Consistent with Sun et al. (Sun et al., 2022), we confirmed a causal correlation between a hereditary disposition to GERD and a risky onset of CAD and did not find any causal presence of CAD on GERD through a bidirectional MR analysis. Additionally, in line with a nationwide cohort study (Kristensen et al.), we discovered that genetic liability to GERD increased the risk of HF. Besides, these two causalities survived after multiple testing and MVMR analysis. |
| 17 | **Generalizability** | Discuss the generalizability of the study results (a) to other populations, (b) across other exposure periods/timings, and (c) across other levels of exposure | Discussion | We detected a series of GDs, of which genetic liability to GERD and celiac disease causally increased the risk of both CAD and HF, together with duodenal ulcer. Our discoveries may shed light on the early recognition and appropriate management of GDs in patients with CAD and HF. |
|  | **OTHER INFORMATION** |  |  |  |
| 18 | **Funding** | Describe sources of funding and the role of funders in the present study and, if applicable, sources of funding for the databases and original study or studies on which the present study is based | Funding | None. |
| 19 | **Data and data sharing** | Provide the data used to perform all analyses or report where and how the data can be accessed, and reference these sources in the article. Provide the statistical code needed to reproduce the results in the article, or report whether the code is publicly accessible and if so, where | - | All exposure and outcome data sources were obtained from public databases, and we have displayed the specific information in the uploaded Appendix 1-Table 1, including Consortium/Author and Web source/Pubmed ids. In addition, to get the code, please email to zgzyzz0919@163.com. |
| 20 | **Conflicts of Interest** | All authors should declare all potential conflicts of interest | **Conflicts of Interest** | The authors declare that there is no conflict of interests in this study. |

This checklist is copyrighted by the Equator Network under the Creative Commons Attribution 3.0 Unported (CC BY 3.0) license.

1. Skrivankova VW, Richmond RC, Woolf BAR, Yarmolinsky J, Davies NM, Swanson SA, et al. Strengthening the Reporting of Observational Studies in Epidemiology using Mendelian Randomization (STROBE-MR) Statement. JAMA. 2021;under review.

2. Skrivankova VW, Richmond RC, Woolf BAR, Davies NM, Swanson SA, VanderWeele TJ, et al. Strengthening the Reporting of Observational Studies in Epidemiology using Mendelian Randomisation (STROBE-MR): Explanation and Elaboration. BMJ. 2021;375:n2233.
